# Structural characterisation of high affinity Siglec-2 (CD22) ligands in complex with whole Burkitt’s lymphoma (BL) Daudi cells by NMR spectroscopy

**DOI:** 10.1038/srep36012

**Published:** 2016-11-03

**Authors:** Paul D. Madge, Andrea Maggioni, Mauro Pascolutti, Moein Amin, Mario Waespy, Bernadette Bellette, Robin J. Thomson, Sørge Kelm, Mark von Itzstein, Thomas Haselhorst

**Affiliations:** 1 Institute for Glycomics, Gold Coast Campus, Griffith University, Queensland, 4222, Australia; 2 Centre for Biomolecular Interactions Bremen, Department of Biology and Chemistry, University of Bremen, 28334 Bremen, Germany

## Abstract

Siglec-2 undergoes constitutive endocytosis and is a drug target for autoimmune diseases and B cell-derived malignancies, including hairy cell leukaemia, marginal zone lymphoma, chronic lymphocytic leukaemia and non-Hodgkin’s lymphoma (NHL). An alternative to current antibody-based therapies is the use of liposomal nanoparticles loaded with cytotoxic drugs and decorated with Siglec-2 ligands. We have recently designed the first Siglec-2 ligands (9-biphenylcarboxamido-4-*meta*-nitrophenyl-carboxamido-Neu5Acα2Me, 9-BPC-4-*m*NPC-Neu5Acα2Me) with simultaneous modifications at C-4 and C-9 position. In the current study we have used Saturation Transfer Difference (STD) NMR spectroscopy to monitor the binding of 9-BPC-4-*m*NPC-Neu5Acα2Me to Siglec-2 present on intact Burkitt’s lymphoma Daudi cells. Pre-treatment of cells with periodate resulted in significantly higher STD NMR signal intensities for 9-BPC-4-*m*NPC-Neu5Acα2Me as the cells were more susceptible to ligand binding because *cis*-binding on the cell surface was removed. Quantification of STD NMR effects led to a cell-derived binding epitope of 9-BPC-4-*m*NPC-Neu5Acα2Me that facilitated the design and synthesis of C-2, C-3, C-4 and C-9 tetra-substituted Siglec-2 ligands showing an 88-fold higher affinity compared to 9-BPC-Neu5Acα2Me. This is the first time a NMR-based binding study of high affinity Siglec-2 (CD22) ligands in complex with whole Burkitt’s lymphoma Daudi cells has been described that might open new avenues in developing tailored therapeutics and personalised medicine.

The vast majority (90%) of non-Hodgkin’s lymphoma (NHL) cases are B cell malignancies. According to the American Cancer Society, it is expected that nearly 71,850 people will be diagnosed with NHL in the United States in 2015, and nearly 19,790 will die from the disease^1^. Although therapy with the anti-CD20 antibody Rituximab (Rituxan^®^ and MabThera^®^) is effective, it is not a cure, especially for the indolent lymphoid malignancies. In addition, 80% of patients treated with Rituximab suffer from severe side effects and in some cases from tumour lysis syndrome^2^. Rituximab relies on complement and antibody dependent cell-mediated cytotoxicity to effect cell killing, since CD20 is statically expressed on the cell surface. Novel therapeutics with an alternative B cell lymphoma targeting mechanism are urgently needed. So far, the search for new NHL therapeutics has been focused on monoclonal antibodies that are in clinical development for the autoimmune disease systemic lupus erythematosus^3^. An *antibody-independent* approach using doxorubicin-loaded liposomes decorated with high-affinity Siglec-2 ligands has been proposed^4^,^5^. Because the B cell specific cell surface receptor Siglec-2 (CD22) undergoes constitutive endocytosis, it is well suited for the efficient delivery of toxins into cells and its use does not rely on the patient’s immune system. Thus, immunotoxins based on anti-Siglec-2 antibodies induce B cell killing by a different mechanism to Rituximab, and Siglec-2 has become a validated target for the treatment of B cell lymphomas. Siglec-2 binds with high preference to α(2,6)-linked *N*-acetylneuraminic acid (Neu5Ac)^6^ such as in the trisaccharide epitope Neu5Acα(2,6)Galβ(1,4)GlcNAc^7^. In contrast, the corresponding monosaccharide methyl glycoside Neu5Acα2Me (**1**) (Fig. 1) has a low binding potency with an *IC*_*50*_ value of 1.4 mM^8^. The addition of a biphenylcarboxamido group at C-9 of the Neu5Ac template (9-BPC-Neu5Acα2Me, **2**) (Fig. 1) increased the overall potency by a factor of 224^8^. Doxorubicin-loaded liposomes decorated with 9-BPC-Neu5Acα(2,3)Galβ(1,4)Glc that target B cell lymphoma were effective in extending life in a xenograft mouse model, however malignant B cell killing was not complete, likely due to insufficient affinity and selectivity of the siglec ligand 9-BPC-Neu5AcαGalβ(1,4)Glc that binds Siglec-2 expressed on B cells^4^. Siglec-2 ligands with improved binding affinity have been developed^9^,^10^ however, our group has succeeded in introducing for the first time functionalities at both C-4 and C-9 positions on **2**, 9-biphenylcarboxamido-4-*meta*-nitrophenyl-carboxamido-Neu5Acα2Me (9-BPC-4-*m*NPC-Neu5Acα2Me, **3**) (Fig. 1) using a NMR-based structure guided approach^11^. Ligand **3** showed a 15-fold higher affinity than the corresponding mono-substituted derivative 9-BPC-Neu5Acα2Me (**2**)^11^.

Here we study the molecular interaction between high affinity Siglec-2 ligands and Siglec-2 based on high-resolution NMR experiments performed in a cellular environment using intact and live BL Daudi cells. Bulky biological macromolecules such as intact viruses, virus-like particles (VLPs) and cells using NMR techniques are ideal targets to monitor ligand binding^12^,^13^,^14^,^15^,^16^,^17^. We, and others, have shown that isolated cell membranes can also be used to investigate the molecular interaction between cellular receptors and bioactive compounds^18^,^19^,^20^,^21^,^22^. In the current study we have analysed the binding of 9-BPC-4-*m*NPC-Neu5Acα2Me (**3**) directly to Siglec-2 expressed on intact BL Daudi cells by Saturation Transfer Difference (STD) NMR spectroscopy. Quantification of STD NMR effects has resulted in a cell-derived binding epitope that guided the design of second-generation high affinity tetra-substituted Siglec-2 ligands 9-BPC-2-(4-carboxymethyl-[1,2,3]triazol-1-yl)-3-hydroxy-4-*meta*-nitrophenyl-carboxamido-Neu5Acα2Me (**7**) and 9-BPC-2-(4-carboxymethyl-[1,2,3]triazol-1-yl)-3-hydroxy-4-*meta*-methoxyphenyl-carboxamido-Neu5Acα2Me (**8**). Ligands **7** and **8** showed significantly improved Siglec-2 binding affinity with r*IP* values of 87.6 and 58.1 respectively, compared to the benchmark compound **2**.

## Results

### Binding of 9-BPC-4-*m*NPC-Neu5Acα2Me to Burkitt’s lymphoma (BL) Daudi cells

**Figure 2:**
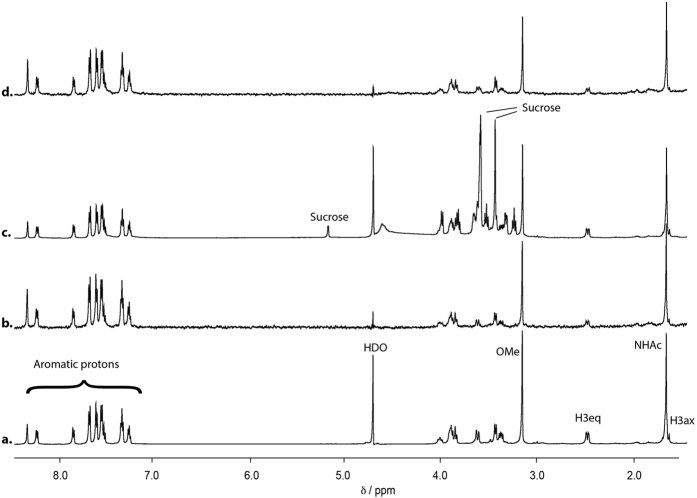
STD NMR of Siglec-2 ligand 3 complexed with BL Daudi cells. STD NMR spectra of 0.5 mM **3** in the presence of 5.0 × 10^5^ BL Daudi cells in 1.5 mM deuterated HEPES, 140 mM NaCl at 283 K, 600 MHz and pH 7.4. The saturation time of 2 s and 256 scans resulting in a total acquisition time of 53 min. On-resonance frequency was set to −1 ppm and the off-resonance to −300 ppm. (**a**) ^1^H and (**b**) STD NMR spectra of **3**; (**c**) ^1^H and (**d**) STD NMR spectra of an equimolar mixture of **3** and the non-binding spy molecule, sucrose. Notably, BL Daudi cells recognise **3** with an identical epitope as recombinant Siglec-2 in solution^11^.

In the first instance, we have monitored the binding of 9-BPC-4-*m*NPC-Neu5Acα2Me (**3**) directly to Burkitt’s lymphoma (BL) Daudi cells using Saturation Transfer Difference (STD) Nuclear Magnetic Resonance (NMR) spectroscopy. STD NMR spectroscopy is an ideal tool to study the interaction of larger-sized biomolecular targets with low-molecular-weight ligands, and has been previously used to investigate ligand interactions with bulky targets, for example whole virus particles^12^,^23^, cells^14^,^16^, sepharose immobilized recombinant protein^24^, virus-like particles^13^,^25^ and intracellular organelles^20^,^21^,^26^. The viability of BL Daudi cells in 2.0 mM deuterated HEPES buffer was assessed by monitoring the cells with increasing sodium chloride concentration. The absence of sodium chloride in the buffer resulted in a 79% decrease in viable cells after 1 h. The addition of either 300 mM, 150 mM or 75 mM sodium chloride to 1.5 mM deuterated HEPES chloride prevented the BL Daudi undergoing osmotic shock with only 2.9%, 1.9% and 2.1% loss in viability, respectively. In order to keep the NMR pulse length short, a buffer with lower salt concentrations (70 and 140 mM) was chosen. The ^1^H NMR spectra of **3** in the presence of 5.0 × 10^5^ BL Daudi cells buffered (1.5 mM deuterated HEPES with 140 mM NaCl) are shown in Fig. 2a; the corresponding STD NMR spectrum (Fig. 2b) reveals strong and significant signals for **3** when bound to the Daudi cells. We have determined the viability of the BL Daudi cells before (95.5%) and after incubation (1 hour at 37 °C and 4 °C) in the NMR buffer (1.5 mM HEPES, 75 mM NaCl, D_2_O). These experiments revealed that 92.5% of BL Daudi cells were still viable after 1 h incubation at 4 °C and 91.2% at 37 °C demonstrating that only a very small amount of cells have died during NMR spectra acquisition. However, cell viability after a 3 h incubation period was 83% at 4 °C and 63% at 37 °C indicating that the cells considerably loose their viability over longer time periods and that only shorter acquisition times can be used.

To investigate if the binding of **3** to the BL Daudi cells is specific we added an equimolar concentration of a non-binding spy molecule (sucrose) and acquired a ^1^H NMR spectrum as shown in Fig. 2c. The STD NMR spectrum of this equimolar **3**:sucrose-mixture is shown in Fig. 2d. Most striking is the observation that no STD NMR signals can be detected for the spy molecule sucrose, demonstrating that it has no binding affinity to the Daudi cells. This result clearly supports the conclusion that the observed STD NMR signals of **3** in the presence of BL Daudi cells are a direct consequence of **3** binding to the cell surface protein.

### 9-BPC-4-*m*NPC-Neu5Acα2Me (3) bound to periodate pretreated BL Daudi cells reveals an increase in relative STD NMR effects

In order to investigate if unmasking of siglecs present the BL Daudi cell surface that are engaged in *cis* interaction would result in more efficient binding and hence stronger STD NMR signals of **3**, BL Daudi cells were pre-treated with periodate that specifically truncates the glycerol side chain of sialic acid of the glycosylated Siglec-2^27^. STD NMR experiment of **3** in complex with pretreated BL Daudi cells has revealed a significant increase in STD NMR signal intensities (Supplementary Figure 1) of **3** presumably due to the disruption of *cis*-binding resulting in an increase in Siglec ligand binding sites on the cell surface^28^.

### 9-BPC-4-*m*NPC-Neu5Acα2Me (3) bound to Siglec^high^ BL Daudi cells reveals higher STD NMR effects compared to Siglec^low^ BL Daudi cell fractions

**Figure 3:**
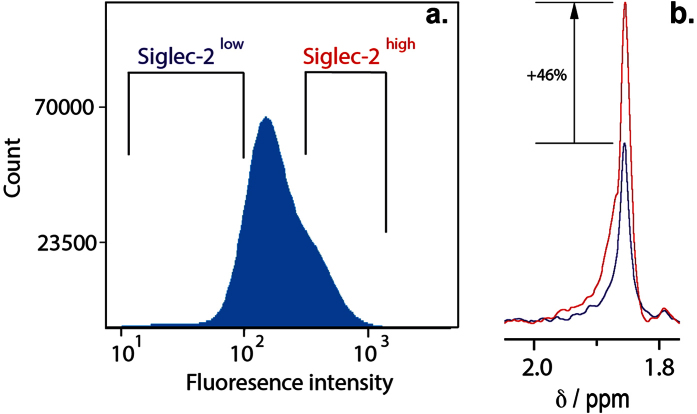
BL Daudi cells stained with BV421 targeting Siglec-2 primary antibody. (**a**) Cells were divided into Siglec-2^low^ and Siglec-2^high^ fractions as indicated based on their mean fluorescence. (**b**) Absolute STD NMR signals of the methyl protons of the *N*-acetamido group of **3** in the presence of 5 × 10^5^ Siglec-2^low^ (blue) and Siglec-2^high^ BL Daudi cells (red). The absolute STD NMR signal intensity showed a 46% increase using the Siglec-2^high^ BL Daudi cell fraction (red).

To further obtain evidence that **3** binds specifically to Siglec-2 expressed on BL Daudi cells we have performed fluorescence activated cell sorting of these cells into Siglec-2^high^ and Siglec-2^low^ fractions. The cells were stained using an anti-human Siglec-2 mAb (clone RFB4) as primary antibody and anti-antibody Brilliant Violet 421 conjugated secondary antibody. Cells were sorted into Siglec-2^low^ and Siglec-2^high^ fractions based on their median fluorescence intensities (Fig. 3a). Siglec-2^low^ and Siglec-2^high^ cell fractions were collected and counted to obtain an identical number of cells (5.0 × 10^5^ BL Daudi cells), transferred into the cell-specific NMR buffer, complexed with **3**, and subjected to STD NMR experiments. Figure 3b shows the absolute STD NMR signals of the methyl protons of the *N*-acetyl group of **3** in the presence of 5.0 × 10^5^ Siglec-2^low^ (blue) and Siglec-2^high^ (red) BL Daudi cells. The spectra reveal that the Siglec-2^high^ cells have a 46% increased STD NMR signal intensity compared to the Siglec-2^low^ cells, correlating with the increased number of available Siglec-2 copies present on the cell surface. This analysis demonstrates that the binding of **3** to BL Daudi cells occurs via Siglec-2 expressed on the cell surface. To ensure that the binding of **3** occurred to Siglec-2 and not to other Siglecs expressed on the cells^29^, BL Daudi cells used in these experiments were stained with a panel of anti-human siglec antibodies and relevant controls. The quantitative analysis (Table 1 and Supplementary Figure 2a) revealed that a low percentage of cells express other Siglecs, eg. 5.9%, 6.7% and 4.5% for Siglec-10, Siglec-3 and Siglec-5/-14, respectively. Only Siglec-11 and Siglec-16 are present in 13.4% and 11.5% of the cells respectively, whereas less than 2% of cells express Siglec-1, -4, -6, -7, -8 and -9. This analysis suggests that the observed binding of **3** to BL Daudi cells is a direct consequence of its interactions with Siglec-2 and cross-reactivity with other Siglecs recognizing α(2,3)-/α(2,6)-Neu5Ac glycosides is negligible.

### Binding of 9-BPC-4-*m*NPC-Neu5Acα2Me (3) to transiently expressed Siglec-2 on HEK293T cells

**Figure 4:**
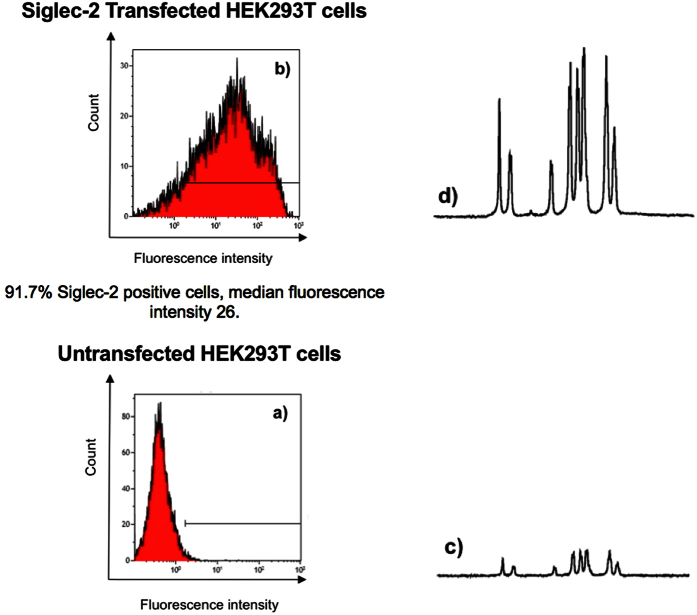
Flow cytometry histograms of untransfected (**a**) and Siglec-2 transfected (**b**) HEK293T cells for the evaluation of Siglec-2 expression using anti-human Siglec-2 antibody. STD NMR spectra of **3** in the presence of 1.0 × 10^6^ untransfected HEK293T cells (**c**) and 1.0 × 10^6^ Siglec-2 transfected HEK293T cells (**d**) at 283 K and 600 MHz. The absolute STD NMR signal intensity showed a 75 times increase for **3** in complex with Siglec-2 transfected HEK293T cells (**d**) compared to untransfected HEK293T cells (**c**). The saturation time for all experiments was 2 s and 256 scans resulting in a total acquisition time of 53 min. On-resonance frequency was set to −1 ppm and the off-resonance to −300 ppm.

To further demonstrate the binding of **3** to Siglec-2 expressed on cells an additional control NMR experiment was performed using HEK293T cells before and after transfection with the full length Siglec-2 coding sequence (Uniprot P20273-1). The quantitative analysis of Siglec expression (Fig. 4a) in untransformed HEK293T cells revealed that a very low percentage of cells express Siglec-2 (1.2% of the population) making this cell line an ideal negative control to study Siglec-2 ligand interactions. Only minimal expression of Siglec-4, Siglec-10, Siglec-11 and Siglec-16 can be detected on HEK293T cells hence, minimizing the contributions coming from the interaction of the ligand of interest with other Siglecs. (Table 1 and Supplementary Figure 2b). The flow cytometric analysis of Siglec-2 transfected HEK293T cells (Fig. 4b) revealed the highest Siglec-2 expression (91.7% positive, median fluorescence intensity 26.9) after 24 hours post transfection using 3.75 μL of Lipofectamine 3000 reagents to prepared DNA-lipid complexes. Non-transfected and Siglec-2 transfected HEK293T cells were then complexed with **3** and STD NMR experiments were undertaken. Very low signal intensity was observed for **3** in complex with non-transfected HEK293T cells (Fig. 4c). An identical NMR experiment performed with Siglec-2 transfected HEK293T cells in complex with **3** showed a 75-fold increase in STD NMR signal intensity correlating with the significantly increased number of available Siglec-2 binding sites present on the HEK293T cell surface (Fig. 4d). This result does not only support that **3** binds specifically to Siglec-2 expressed on the cell surface, but also that a higher protein expression level leads to an increased STD NMR signal intensity due to the availability of an increased number of binding sites.

### Binding epitope of 3 bound to BL Daudi cells

**Figure 5:**
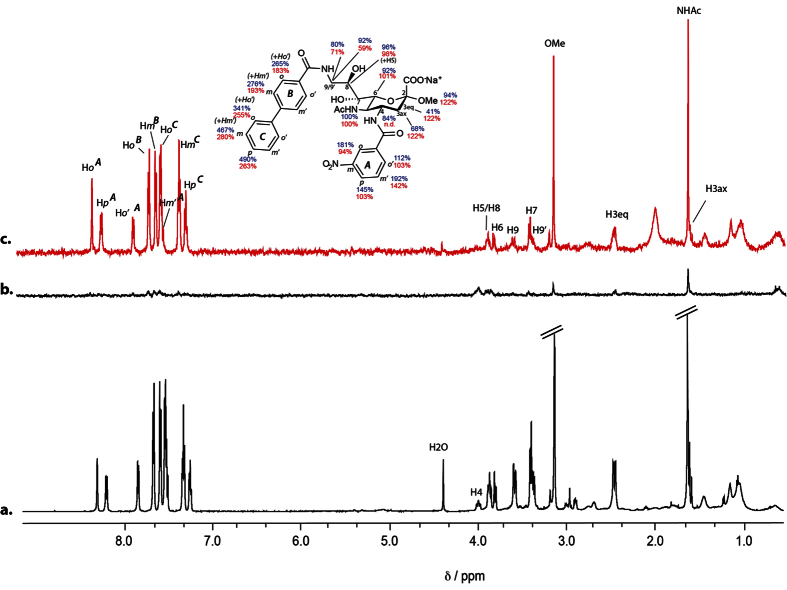
STD NMR of Siglec-2 ligand 3 complexed with BL Daudi cells. STD NMR spectra of 0.5 mM **3** in the presence of 5.0 × 10^5^ BL Daudi cells in 1.5 mM deuterated HEPES, 140 mM NaCl at 283 K, 600 MHz and pH 7.4. The saturation time of 2 s and 256 scans resulting in a total acquisition time of 53 min. On-resonance frequency was set to −1 ppm and the off-resonance to −300 ppm. (**a**) ^1^H and (**b**) STD NMR of **3** in the absence of protein or cells (**c**), STD NMR of **3** in the presence of 5.0 × 10^5^ BL Daudi cells (red). The relative STD NMR effects of **3** in the presence of cells (red values) are shown. The binding epitope was calculated using a double difference (STDD) NMR spectrum by subtracting the control spectrum obtained in the absence of cells b) from the spectrum acquired for the **3**-cell complex. STD NMR effects derived from **3** in complex with Siglec-2 (blue values) were taken from published values^11^.

Our structural approach comprises of the quantitative evaluation of STD NMR effects of **3** when bound to BL Daudi cells and to generate a binding epitope that consequently facilitate the design of second-generation Siglec-2 ligands. First, we were interested to compare the relative STD NMR values of **3** in complex with BL Daudi cells to our recently determined STD NMR values obtained for **3** bound to recombinantly-expressed Siglec-2^11^, The binding epitope of **3** in complex with BL Daudi cells was determined by double difference STD (STDD) NMR spectroscopy (Fig. 5c) and compared to published relative STD NMR values obtained for a **3**-Siglec-2 complex (red and blue STD NMR values in Fig. 5, respectively). Remarkably, the overall relative STD NMR effects of the **3**-Siglec-2 and **3**-BL Daudi cells complexes are comparable suggesting an identical binding mode. The BPC moiety at C-9 and the *m*NPC ring at C-4 show in both complexes the strongest saturation transfer. The STD NMR spectra of **3** in the presence of BL Daudi cell further revealed that the methyl protons of the C-2 aglycon methoxy group in **3** receive the same level of saturation as the C-5 *N*-acetyl functionality indicating significant interaction of the C-2 aglycon moiety with Siglec-2 expressed on BL Daudi cells. This cell-derived binding epitope is therefore in excellent agreement with the relative STD NMR values obtained for **3** in complex with recombinantly-expressed Siglec-2 suggesting that incorporation of bulkier groups of C-2 aglycon moiety may increase protein contacts and hence further enhance binding affinity. This is also consistent with previous studies that have shown that aromatic moieties at the anomeric (C-2) position, such as a α-benzyl glycoside^30^ or α-2′,3′-dichlorobenzyl^10^ glycosides, of Neu5Ac-based ligands are well tolerated by the binding site and result in enhanced binding affinities. Our cell-derived epitope maps prompted us to introduce a longer alkyl chain. We have also sought to place a carboxylate group directed away from the anomeric position of the Neu5Ac-based ligand, whilst maintaining an aromatic functionality which mimics the interaction of the galactose moiety as present in the natural ligand Neu5Acα(2,6)Galβ(1,4)GlcNAc. Due to the presence of aromatic groups at both C-9 and C-4 of the Neu5Ac-based ligand scaffold, an increase in hydrophilicity is required while maintaining the pharmacophore aromatic character. Therefore, a functionalized [1,2,3]-triazole ring at the anomeric position was introduced for the first time with a carboxymethyl moiety present on the triazole ring to moderate hydrophilicity and to potentially improve the binding affinity. The strong STD NMR effect of the H3 equatorial proton (H3eq) of **3** when bound to BL Daudi cells (122%, red values, Fig. 5) also indicated a pivotal role in binding. This result has promoted us to incorporate a C-3-hydroxyl group. The relative STD NMR effects determined for the *H*^*o*^ and *H*^*p*^protons (ring A) of **3** in complex with BL Daudi cells (94% and 103%, respectively; red values, Fig. 5) suggest that an integration of a hydrophobic group at the *meta* position of ring A might enhance protein contacts and consequently binding affinity.

### Synthesis of second-generation Siglec-2 binding ligands 7 and 8

**Figure 6:**
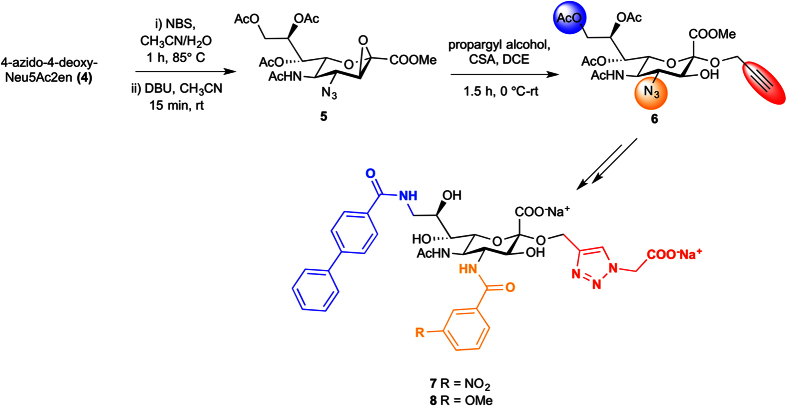
Preparation of 7 and 8.

The synthetic approach towards **7** and **8** commenced with the preparation of 2,3-β-epoxy 4-azido-4-deoxy-Neu5Ac derivative **5**^31^ that is readily accessible from the corresponding 2,3-unsaturated 4-azido-4-deoxy-Neu5Ac2en derivative **4**. Following our recently developed method for accessing 3-hydroxy-Neu5Ac α-glycosides^32^, the key synthetic intermediate 3-hydroxy-2-α-propargyl-Neu5Ac **6** was obtained through an acid catalysed α-stereoselective opening of epoxide **5** (Fig. 6). To our knowledge, this is the first report of a high yielding reaction generating α-glycosides from 2,3-β-epoxy 4-azido-4-deoxy-Neu5Ac (**5**). This method offers great potential for accessing 4-azido-4-deoxy-3-hydroxy-Neu5Ac α-glycosides and could be used to introduce a range of functionalities at the anomeric position to explore interactions with biologically important sialic acid-recognizing proteins.

The presence of a C-3-hydroxyl group in *N*-acetylneuraminic acid derivatives has been shown to increase resistance to enzymatic hydrolysis by sialidases^33^ and, in some cases, may yield slight improvements in binding affinity compared to the unsubstituted parent compound^9^. As shown in Fig. 6, intermediate **6** offers promising opportunities for selective derivatisation at the C-2, C-4 and C-9 positions. Employing our established chemistry^11^, the biphenylcarboxamido group at C-9 and aromatic amide at C-4 were introduced. The aromatic-like moiety at C-2 was then added via a 1,3 dipolar cycloaddition of methyl 2-azidoacetate [CuSO_4_•5H_2_O, sodium ascorbate, MeOH, rt, 2 h] to produce the desired derivatives, which upon base-catalysed deprotection [aq. NaOH, MeOH, rt, 2 h] led to the final compounds **7** and **8**.

### Binding of 7 and 8 to Daudi Burkitt’s lymphoma cells

**Figure 7:**
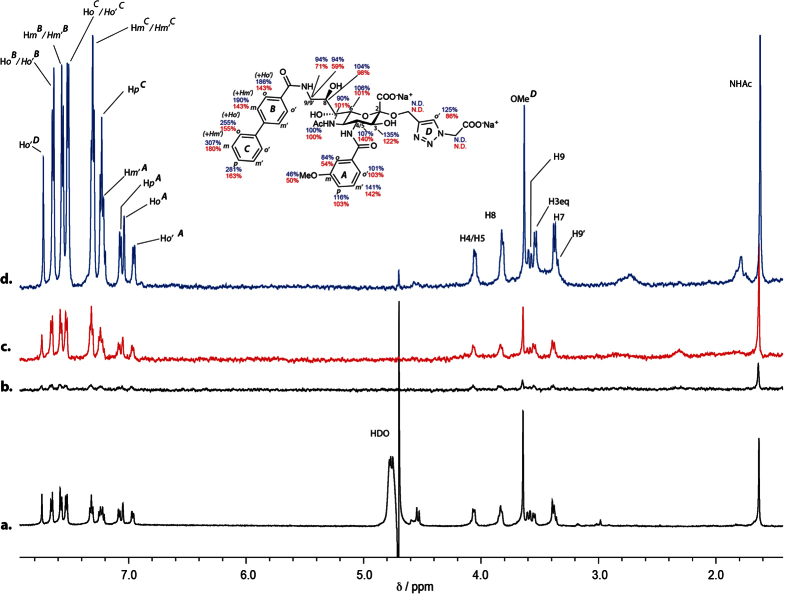
STD NMR of Siglec-2 ligand **7** complexed with BL Daudi cells. (**a**) ^1^H NMR of **7**, (**b**) STD NMR of **7** in the absence of protein or cells (**c**), STD NMR of **7** in the presence of 5.0 × 10^5^ BL Daudi cells (red). (**d**) STD NMR of **7** in the presence of Siglec-2 (blue). The conditions for the cell experiments were 1.5 mM deuterated HEPES, 140 mM NaCl and the ligand concentration was 0.5 mM. STD NMR spectra were acquired at 283 K, 600 MHz and pH 7.4 with a saturation time of 2 s and 256 scans resulting in a total acquisition time of 53 min. On-resonance frequency was set to −1 ppm and the off-resonance to −300 ppm. The relative STD NMR effects of **7** in the presence of cells (red values) and recombinantly expressed Siglec-2 (blue values) are depicted at the molecular structure. The binding epitope was calculated using a double difference (STDD) NMR spectrum by subtracting the control spectrum obtained in the absence of cells b) from the spectrum acquired for the **7**–cell complex c). The control experiment of cells in the absence of ligand did not show any significant background signals and was therefore not used in the quantitative analysis.

**Figure 8:**
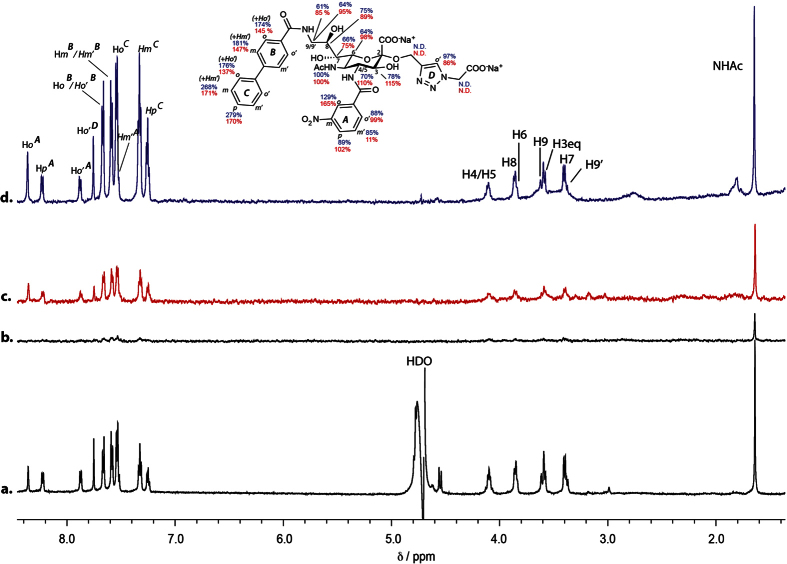
STD NMR of Siglec-2 ligand **8** complexed with BL Daudi cells. (**a**) ^1^H NMR of **8**, (**b**) STD NMR of **8** in the absence of protein or cells (**c**), STD NMR of **8** in the presence of 5.0 × 10^5^ BL Daudi cells (red). (**d**) STD NMR of **8** in the presence of Siglec-2 (blue). The conditions for the cell experiments were 1.5 mM deuterated HEPES, 140 mM NaCl and the ligand concentration was 0.5 mM. STD NMR spectra were acquired at 283 K, 600 MHz and pH 7.4 with a saturation time of 2 s and 256 scans resulting in a total acquisition time of 53 min. On-resonance frequency was set to −1 ppm and the off-resonance to −300 ppm. The relative STD NMR effects of **8** in the presence of cell (red values) and recombinantly expressed Siglec-2 (blue values) are depicted at the molecular structure. The binding epitope was calculated using a double difference (STDD) NMR spectrum by subtracting the control spectrum obtained in the absence of cells b) from the spectrum acquired for the **8**–cell complex (**c**).

We then investigated the binding of **7** and **8** directly on B lymphoblast cells. Figure 7a shows the ^1^H NMR spectrum of **7** and the corresponding STD NMR spectrum of **7** in complex with BL Daudi cells is shown in Fig. 7c. The corresponding STD NMR spectrum of **7** in complex with recombinantly-expressed Siglec-2 is shown in Fig. 7b,d depicts a control STD NMR spectrum of **7** in the absence of both cells and protein. It is immediately obvious that strong STD NMR signals result when **7** is in complex with Daudi cells. A comparison with the STD NMR spectrum of **7** in complex with recombinantly-expressed Siglec-2 (Fig. 7d) indicates similar signal intensities. We have quantified the relative STD NMR signals of **7** when bound to BL Daudi cells (red) and pure Siglec-2 (blue) as shown on the molecular structure of **7**. Remarkably, the binding epitope of **7** is very similar, if not, identical when comparing protein-based and cell-based NMR spectra. This result demonstrates that **7** presumably binds to Siglec-2 expressed on the BL Daudi cell surface. We have performed a similar analysis of the binding epitope of **8** in complex with Siglec-2 and BL Daudi cells (Fig. 8). Figure 8c,d show the STD NMR spectra of **8** in complex with BL Daudi cells or recombinantly-expressed Siglec-2, respectively. The experimentally determined relative STD NMR values and binding epitope revealed very similar values indicating that **8** binds almost exclusively to Siglec-2 expressed on BL Daudi cells.

### Binding affinity of 7 and 8 to recombinantly-expressed Siglec-2 using a hapten inhibition assay and Surface Plasmon Resonance

**Figure 9:**
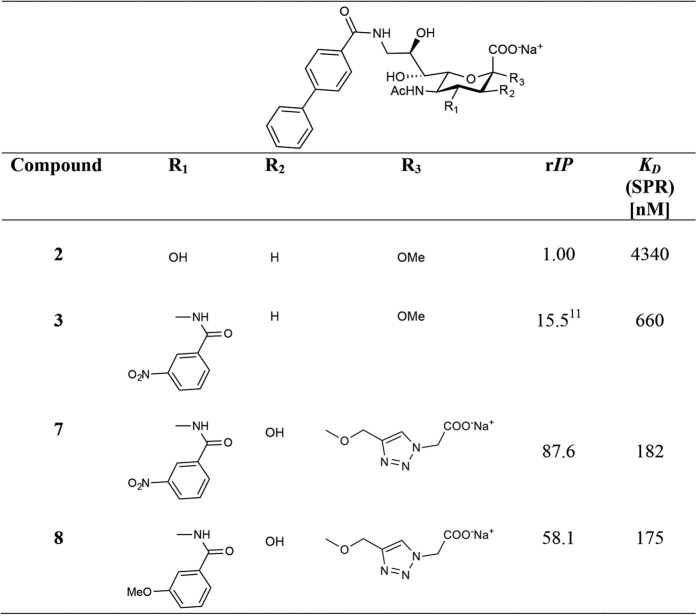
r*IP* values of C-2/C-3/C-4/C-9 modified *N*-acetylneuraminic acid derivatives for Siglec-2. r*IP* values were calculated using 9-BPC-Neu5Acα2Me (**2**) as 1.00. Compound **7** and **8** with an additional C-2 substituent (R_3_) reveal an increase in affinity of 87.6 and 58.1, respectively.

Figure 9 outlines the inhibition potencies of compounds **2**, **3**, **7** and **8** as determined by hapten inhibition assays. The addition of a *meta*-nitrophenylcarboxamido (*m*NPC) moiety at C-4 with a biphenylcarboxamido (9-BPC) at C-9 (**3**) shows enhanced binding to Siglec-2 by a factor of 15.9 compared to the benchmark compound **2** (Fig. 9). The hapten inhibition assay for **7** that bears an additional triazole-carboxy moiety at C-2 and the hydroxyl group at C-3 revealed a relative *IC*_*50*_ (*rIP*) of 86 compared to the benchmark compound **2**. The *rIP* of compound **8** was 58 compared to **2**. Absolute binding affinities were also determined using Surface Plasmon Resonance (SPR) measurements. Dissociation constants (*K*_*D*_) were obtained for **7** and **8** (*K*_*D*_ = 182 nM and *K*_*D*_ = 175 nM, respectively) (Fig. 9 and Supplementary Figure 3).

## Discussion

In the current study, we have demonstrated the binding of high-affinity Siglec-2 ligands directly to BL Daudi cells using NMR spectroscopy. Our NMR-derived results suggest that ligand binding occurs exclusively to Siglec-2 present on BL Daudi cells. Control NMR experiments using HEK293T cells that naturally express Siglec-2 at a very low level revealed very weak ligand STD NMR signals, whereas Siglec-2 transfected HEK293T cells showed a significant increase due to the availability of an increased number of Siglec-2 binding site. In an additional control experiment, by spiking the ligand-BL Daudi cell complex with a non-binding spy molecule sucrose, we have shown that sucrose does not bind to the cells and that therefore the binding of the ligand is specific to Siglec-2 expressed on the BL Daudi cells. The likelihood that the synthesised *N*-acetylneuraminic acid-based ligands also bind to other Siglecs (e.g. Siglec-1, -3, -4, -5, -6, -7, -8, -9, -10, -11, -14 and -16) present on BL Daudi cells is remote as they are present in low populations (Table 1). In addition, STD NMR data for 9-BPC-4-*m*NPC-Neu5Acα2Me (**3**) in the presence of the Siglec-2^high^ cell fraction clearly revealed significantly higher STD NMR signal intensity due to the presence of an increased number of Siglec-2 ligand binding sites. Interestingly, our previously reported analysis of *N*-acetylneuraminic acid derivatives in complex with various Siglecs revealed that binding to different Siglecs resulted in distinct binding epitopes^11^. The cell-derived binding epitope of **3** presented in the current study was similar to the ligand in complex with recombinant protein and was used to guide the synthesis of **7** that showed a 88-fold affinity in binding affinity. The close proximity of the equatorial proton at C-3 to the protein surface led to the introduction of a C-3 hydroxyl group. Strong relative STD NMR effects of *H*^*o*^ and *H*^*p*^ of **3** adjacent to the *meta*-nitro group in ring A have led to the introduction of a *meta*-methoxy group. Finally, STD NMR signals revealed the importance of the C-2 methoxy aglycon moiety of **3** and a more bulkier functionalized [1,2,3]-triazole ring was introduced. Overall, we have shown that the use of whole BL Daudi cells in NMR binding experiments is a straightforward approach and can add invaluable structural information to the design process of second-generation high-affinity ligands. We have also demonstrated for the first time disruption of cis-binding directly at cell-level by NMR spectroscopy.

In conclusion, our whole-cell NMR approach does not require cloning, expression and purification of the target protein and NMR experiments can be acquired in less than an hour. This is the first time a STD NMR-based study of high affinity Siglec-2 ligands directly at cell level has been described and the approach, in general, provides new avenues in the development of tailored therapeutics and personalised medicines. Specifically, our approach could enable the direct assessment of ligand and inhibitor affinity using patient-derived cells in a fast and efficient manner. Moreover, the obtained structural information could be utilised to design novel inhibitors and potential drugs with higher affinity and efficacy. A few challenges in the preparation of samples for cell-based NMR experiments remain. Samples of high homogeneity are essential and cells need to survive the acquisition time, often in non-optimal buffer conditions suitable for NMR acquisition. However, with further development of cryogenic probe technology, reduced NMR acquisition time will offer an excellent platform for structure-guided inhibitor design at cell level.

## Methods

### Burkitt’s lymphoma (BL) Daudi and HEK293T cells culture

The BL Daudi cell line was purchased from the European Collection of Cell Cultures, Public Health England, through Sigma Aldrich (St. Louis, MO, USA). The HEK293T cells were purchased from ATCC (Manassas, VA, USA). The cells were routinely maintained respectively in RPMI-1640 and DMEM supplemented with 2 mM stable glutamine, 12.5 mM HEPES (Lonza, Basel, Switzerland), 10% FCS (Ausgenex, Qld, Australia), and Normocin (InvivoGen, San Diego, CA, USA) at 37 °C in a humidified atmosphere containing incubator 5% CO_2_/95% air. Cells were free from *Mycoplasma spp*. contaminations at all times, as verified using the Mycoalert Mycoplasma Detection Kit and Lucetta luminometer (Lonza, Basel, Switzerland). Cells were authenticated by CellBank Australia (Children’s Medical Research Institute, Westmead, NSW, Australia) according to the ANSI/ATCC ASN-0002-2011 standard.

### Cell viability assays

Cell viability was assayed using Muse Count & Viability Assay Kit and the Muse Cell Analyser (Merck Millipore, MA, USA). Several deuterated buffers were tested to determine the viability of cells. 140 mM NaCl, 1.5 mM deuterated HEPES in D_2_O was chosen as the ideal NMR buffer as it offered minimal ionic concentration and reduced cell death while maintaining a pH of 7.5.

### Flow cytometry experiments

Anti-human Siglec-1 (CD169)-FITC, (clone: 7-239); Siglec-3 (CD33)-FITC, (clone: AC104.3E3); Siglec-5 (CD170)-FITC (clone: 1A5); Siglec-7 (CD328)-VioBlue, (clone: REA214); Siglec-8-FITC, (clone: 7C9) and relevant isotype controls were purchased from Miltenyi Biotech (Bergisch Gladbach, Germany, UE). Mouse anti-human Siglec-2 (CD22) primary antibody (clone RFB4) and the relevant isotype control (clone 1E2.2) were purchased from Merck Group (Darmstadt, Germany, UE). The rat anti-mouse secondary antibody BV421 conjugated was purchased from BD Biosciences (San Jose, CA, USA). Rabbit anti-human Siglec-4 (Myelin Associated Glycoprotein, MAG) was purchased from LifeSpan BioSciences (Seattle, WA, USA). The Donkey anti-rabbit secondary antibody (Clone Poly4064)-BV421 conjugated was purchased from Biolegend (San Diego, CA, USA). Anti-human Siglec-5/Siglec-14-APC (Clone 194128), Siglec-6 (CD327)-APC (Clone 767329), Siglec-9-FITC (Clone 191240); Goat Siglec-10-PE Affinity Purified PAb, Goat Siglec-11-PE Affinity Purified PAb, Goat IgG-PE control, Siglec-16-APC (Clone 706022), and relevant isotypes were purchased from R&D Systems (Minneapolis, MN, USA). BL Daudi and HEK293T cells were incubated with the antibodies for one hour at 4 °C in PBS supplemented with 1% FCS (PBS-FBS) at a ratio of 1 μg of antibody per 1 × 10^6^ cells. The cells were washed three times and then suspended in 1 mL of PBS-FBS buffer prior to flow cytometry analysis. Propidium iodide was used at a concentration of 10 ug/mL to differentiate viable from non-viable cells. Secondary antibodies were used according to the suppliers’ instructions. A CyAn ADP Flow Cytometer and Kaluza Flow Cytometry software were used for flow cytometric analysis (Beckman Coulter, Brea, CA, USA). For sorting the BL Daudi cells into Siglec-2^low^ and Siglec-2^high^ fractions, antibody staining was achieved as described above. RPMI-1640 medium containing 50% v/v FBS was used for collecting sorted cells to minimise cell damage. Cells were sorted into low and high expressing Siglec-2 groups based on their fluorescence intensity, using MoFlo XDP (Beckman Coulter, CA, USA).

### NMR experiments using BL Daudi and HEK293T cells

STD NMR spectra were performed using a Bruker Advance 600 MHz spectrometer, equipped with a 5-mm TXI probe with triple axis gradients at 283 K. 5 × 10^5^ BL Daudi and 1 × 10^6^ HEK293T cells were washed three times with the optimised deuterated NMR buffer by centrifugation at 1,300 rpm, at room temperature for 5 min and resuspending in 200 μL. The ligand concentration was adjusted to 0.5 mM for all experiments. To determine the binding epitope of the ligands when bound to cells STDD (Saturation Transfer Double Difference) spectra were generated by subtracting STD NMR signals from the ligand-cell complex from the spectrum obtained in the absence of cells or protein.

### Transfection of HEK293T cells with Sigle-2 coding sequence

The full-length codon-optimized coding sequence of Siglec-2 (Uniprot entry P20273-1) cloned in plasmid pD2517 was purchased from DNA2.0 (Menlo Park, CA, USA). Plasmid amplification was performed by transformation into chemically competent E. coli NEB 5-alpha (Ipswich, MA, USA) followed by purification using a NucleoBond XTRA EF Plasmid Purification Kit (Machery Nagel, Duren, Germany, EU). HEK293T transfection was performed using Lipofectamine 3000 Transfection Reagent Kit (Thermo Fisher Scientific, Carlsbad, CA, USA) according to the supplier’s standard two-steps optimization protocol in Tissue Culture treated 6-wells plates (Corning, Corning, NY, USA) using 500 ng of purified plasmid per well. Transfection efficiency was monitored by flow cytometry analysis 24 and 48 h post-transfection. Briefly, the cells were detached with PBS supplemented with EDTA at a final concentration of 5 mM and subsequently stained with mouse anti-human Siglec-2 primary antibody (clone RFB4) and anti-mouse secondary antibody BV421 conjugated.

### Periodate treatment of BL Daudi cells

Mild periodate treatment to disrupt *cis*-binding was performed following published procedures^28^. BL Daudi cells were resuspended in phosphate buffer (pH 7.4) containing freshly dissolved 2 mM NaIO_4_ and incubated at 4 °C in the dark. After 30 min, the excess of periodate was quenched by adding 10 μL of 20% deuterated glycerol followed by immediate washing with deuterated buffer optimised for cell NMR experiments. The STD NMR on-resonance frequency was set to −1.00 ppm and the off-resonance to 300 ppm. To ensure the viability of the cells during NMR acquisition 256 scans were chosen resulting in a total acquisition time of 53 min.

### Synthesis of Siglec-2 ligands

The *N-*acetylneuraminic acid derivatives **7** and **8** and intermediates were prepared according to Fig. 6. Experimental protocols, a detailed scheme, ^1^H and ^13^C NMR spectra for all compounds are provided in the Supplementary Material.

### Production of recombinant Siglecs

Recombinant Siglec-2 (CD22) Fc-chimeras were expressed in CHO-Lec1 and protein purification was performed as described^34^. The recombinant Siglec-2-Fc chimera, in which the three *N*-terminal Ig-domains of Siglec-2 were fused to the C-terminal human IgG1 Fc domain, was expressed in CHO-Lec1 cells.

### Hapten inhibitions assays

Hapten inhibition assays were performed following published procedures^34^. In brief: Fetuin was used as target in hapten inhibition assays, since Siglec-2 binds to this glycoprotein containing α(2,3)- and α(2,6)-linked *N*-acetylneuraminic acids. The assays were performed in 384 wells microtitre plates determining siglec-Fc binding to immobilized fetuin in the presence of increasing concentrations of compounds to be tested as competitive inhibitors. Specific binding was obtained by subtracting unspecific binding to immobilized desialylated fetuin as target. The half maximal inhibitory concentration of siglec binding (*IC*_*50*_) was determined from corresponding binding curves. Average *IC*_*50*_values and corresponding standard deviations were calculated from these experiments. Each sample was measured in triplicates and each experiment was repeated at least three times. Standard deviations for all compounds were below 15% of the average *IC*_*50*_values. For a better comparison between different assays, for each sample relative *IC*_*50*_ (r*IP*) values were calculated.

### Surface plasmon resonance (SPR) assays

Siglec-Fc chimeras were either captured to anti-Fc antibody-derivatised dextran surface of a Reichert SR7500DC flow cell or directly immobilized by amide coupling. *N*-acetylneuraminic acid derivatives at various concentrations in PBS were passed over the flow cell at a flow rate of 20 μL/min followed by buffer for dissociation. Specific binding was determined by subtracting the signal obtained from the reference cell (ethanolamine derivatised dextran surface) from the sample cell derivatised with Siglec-2 Fc chimeras. The data were analysed and fitted using the software Scrubber (BioLogic). Corresponding sensorgrams for **7** and **8** are shown in Supplementary Figure 2a,b, respectively.

## Additional Information

**How to cite this article**: Madge, P. D. *et al.* Structural characterisation of high affinity Siglec-2 (CD22) ligands in complex with whole Burkitt’s lymphoma (BL) Daudi cells by NMR spectroscopy. *Sci. Rep.* **6**, 36012; doi: 10.1038/srep36012 (2016).

**Publisher’s note**: Springer Nature remains neutral with regard to jurisdictional claims in published maps and institutional affiliations.

## Supplementary Material

Supplementary Information

## Figures and Tables

**Figure 1:**
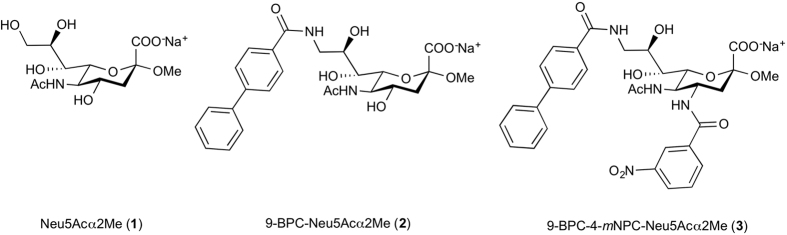
*N*-acetylneuraminic acid derivatives (R = NHAc).

**Table 1:** Siglec expression profile in BL Daudi and HEK293 cells.

Siglec	BL Daudi		HEK293T	
	Positive Population [%]	Median Fluorescence Intensity	Positive Population [%]	Median Fluorescence Intensity
Siglec-1	1.9	1.1	1.1	1.9
Siglec-2	88	9	1.2	2.1
Siglec-3	6.7	1.1	0.8	2.1
Siglec-4	1.3	0.9	27	3
Siglec-5/Siglec-14	4.5	1.3	6.5	2.9
Siglec-6	1.1	1.2	1.9	2.6
Siglec-7	0.9	0.9	1.1	1.2
Siglec-8	0.6	1.7	1.3	2.1
Siglec-9	1.0	1.7	1.1	2.1
Siglec-10	5.9	2.1	1.7	2
Siglec-11	13.4	4.9	10.8	2.9
Siglec-16	11.5	1.6	22.9	4

BL Daudi cells were stained with a panel of anti-human Siglec antibodies and relevant controls and isotypes. The percentage of the positive population for each Siglec is reported together with the corresponding median fluorescence intensity.
