# Supplementary material for: Structural characterisation of high affinity Siglec-2 (CD22) ligands in complex with whole Burkitt’s lymphoma (BL) Daudi cells by NMR spectroscopy
Source: Sci Rep. 2016 Nov 3;6:36012. doi: 10.1038/srep36012 (PMC5093622; doi:10.1038/srep36012)
Supplement: Supplementary Information [file srep36012-s1.pdf]

Structural characterisation of high affinity Siglec-2 (CD22) ligands in complex with whole Burkitt's lymphoma (BL) Daudi cells by NMR spectroscopy

Paul D. Madge[1], Andrea Maggioni[1], Mauro Pascolutti[1], Moein Amin[1], Mario Waespy[2], Bernadette Bellette[1], Robin J. Thomson[1], Sørge Kelm[1,2], Mark von Itzstein[1] and Thomas Haselhorst[1]*

Supplementary figures

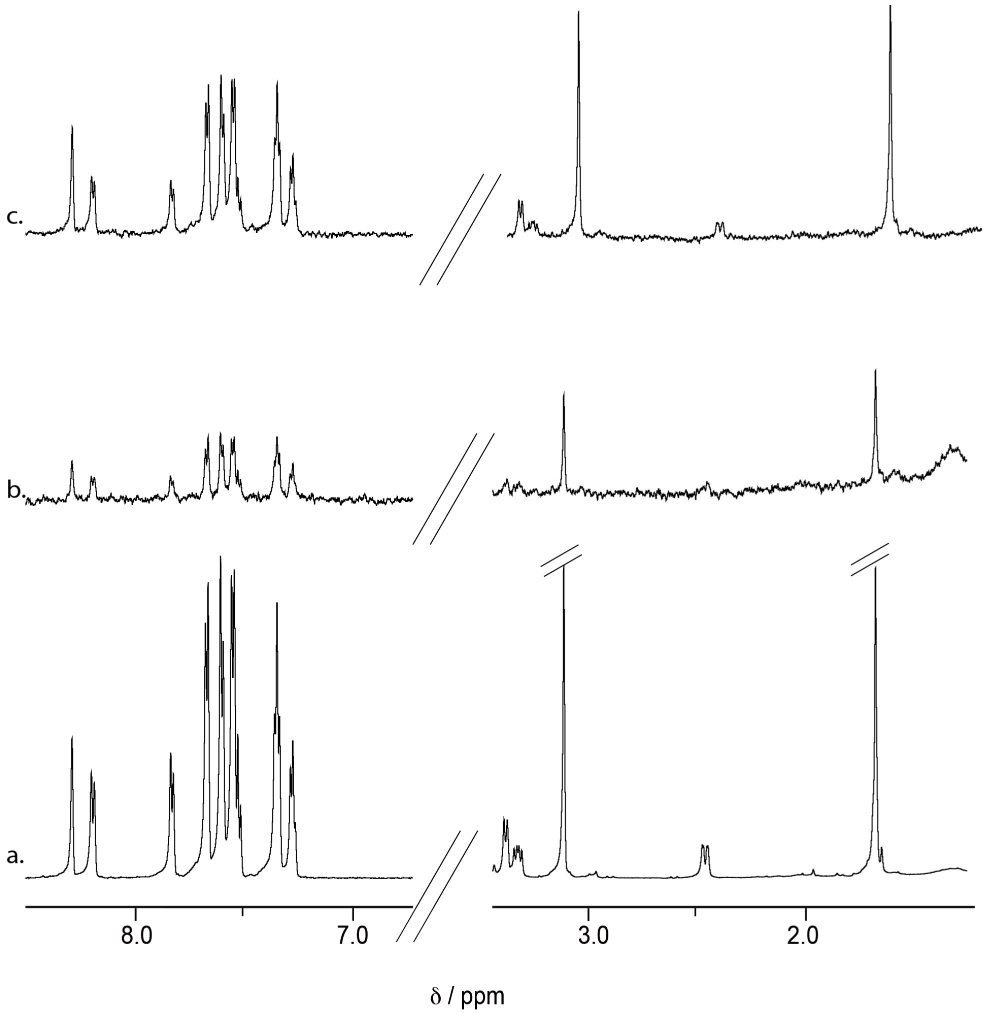

Supplementary Fig. 1. ^{1}H NMR spectrum (**a**) and STD NMR spectra of **3** in the presence of 5.0 ×10[5] BL Daudi cells (**b**) and 5.0 ×10[5] BL Daudi cells pre-treated with periodate (**c**) at 283 K and 600 MHz. The cells were treated with freshly prepared 2 mM $NaIO_4$ and incubated for 30 min at 4 °C in the dark. Excess periodate was quenched by adding 10 µl of 20% deuterated glycerol followed by immediate washing with the deuterated NMR buffer (1.5 mM HEPES, 140 mM NaCl). The saturation time of 2 s and 256 scans resulted in a total acquisition time of 53 min. On-resonance frequency was set to -1 ppm and the off-resonance to -300 ppm.

a)

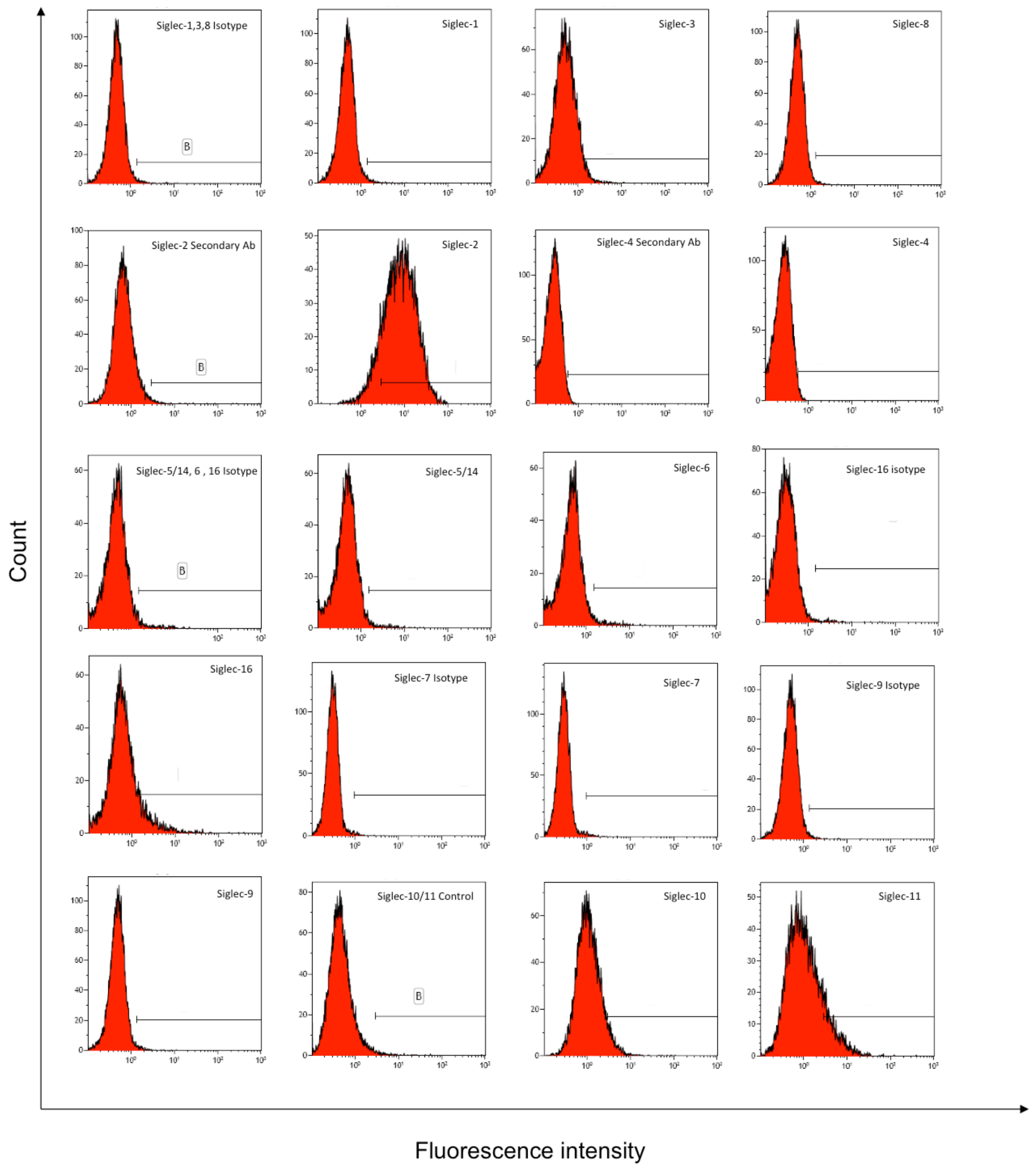
Count
Fluorescence intensity
Siglec-1,3,8 Isotype
Siglec-1
Siglec-3
Siglec-8
Siglec-2 Secondary Ab
Siglec-2
Siglec-4 Secondary Ab
Siglec-4
Siglec-5/14, 6 , 16 Isotype
Siglec-5/14
Siglec-6
Siglec-16 isotype
Siglec-16
Siglec-7 Isotype
Siglec-7
Siglec-9 Isotype
Siglec-9
Siglec-10/11 Control
Siglec-10
Siglec-11

b)

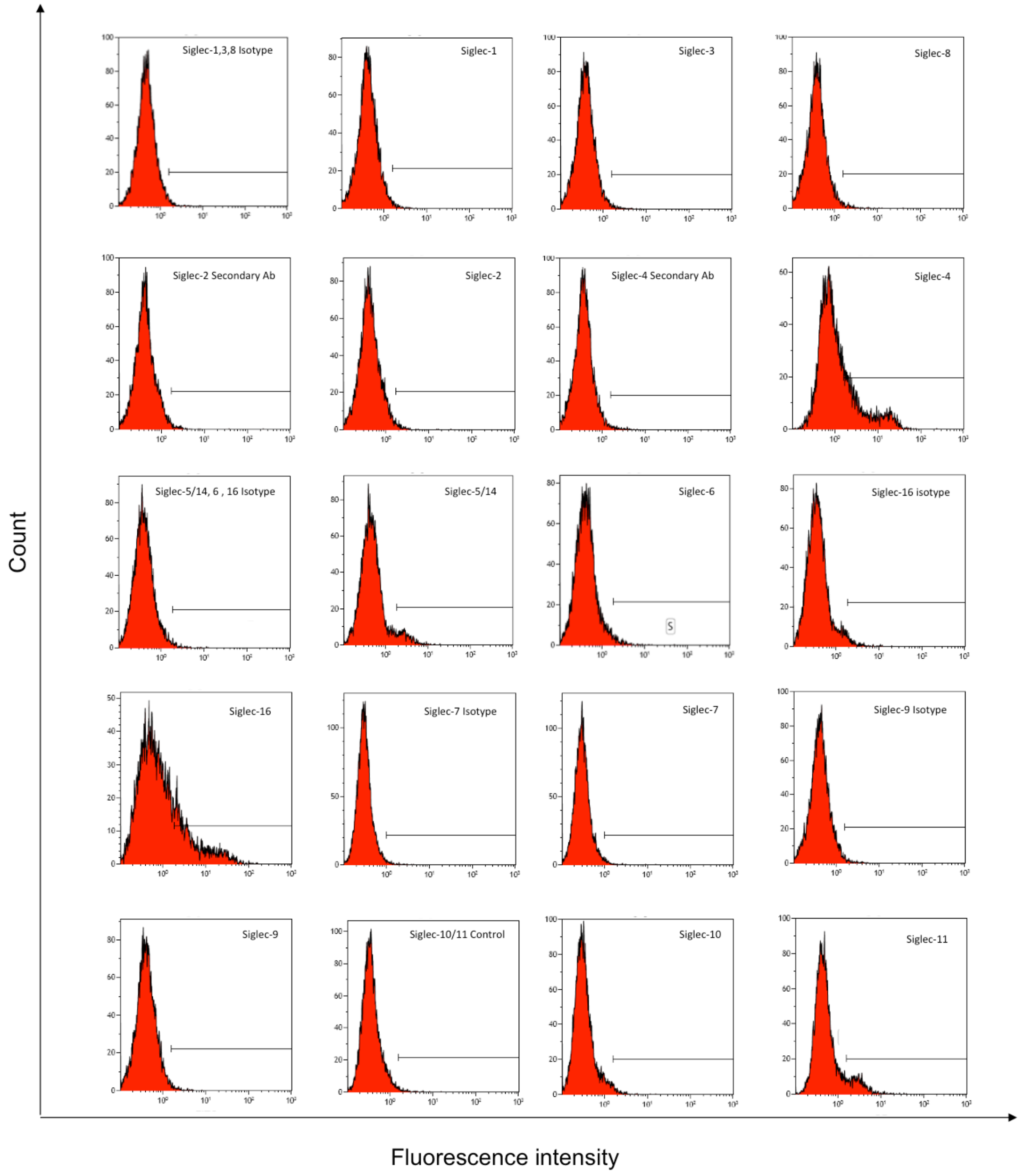

Supplementary Fig.2: Flow cytometry experiments of **a)** Burkitt's lymphoma (BL) Daudi cells and **b)** HEK293T for the evaluation of Siglec expression on the cell membrane. Cells were stained with a panel of anti-human Siglecs antibodies or and the relevant isotype or controls and analysed on a CyAn Flow Cytometer (Beckamn Coulter). 10,000 events were acquired and data were analysed with Kaluza Flow Cytometry Analysis software. Goat IgG-PE for Goat Siglec-10-PE affinity purified PAb and Goat Siglec-11-PE affinity purified PAb were used for Siglec-10/11

controls. Supplementary Table 1 summarises the population expressed.

a)

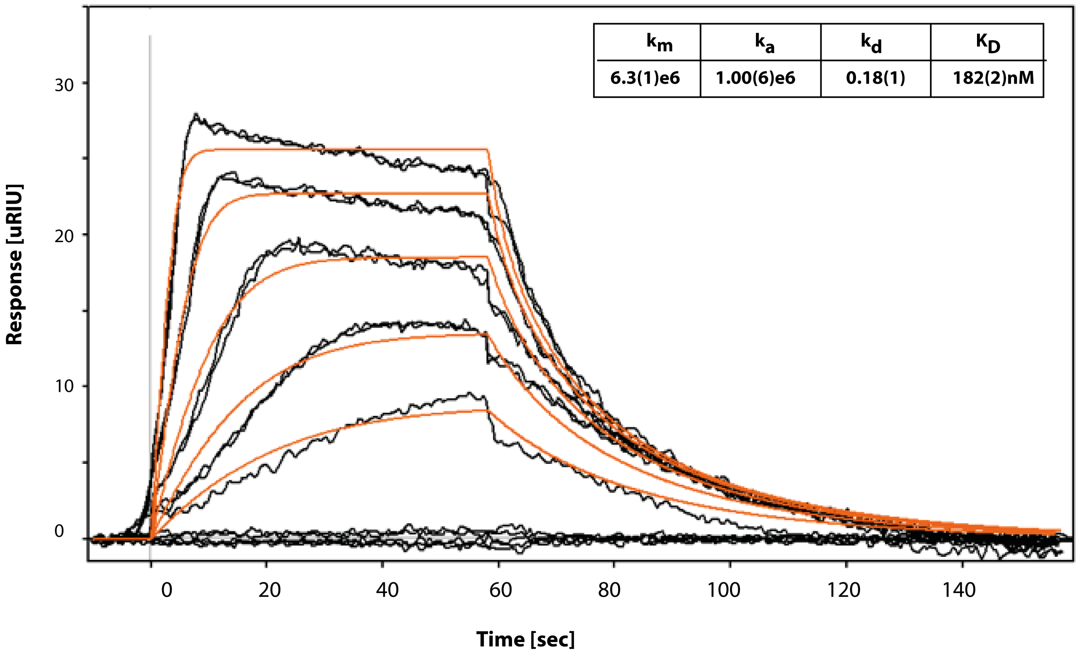

b)

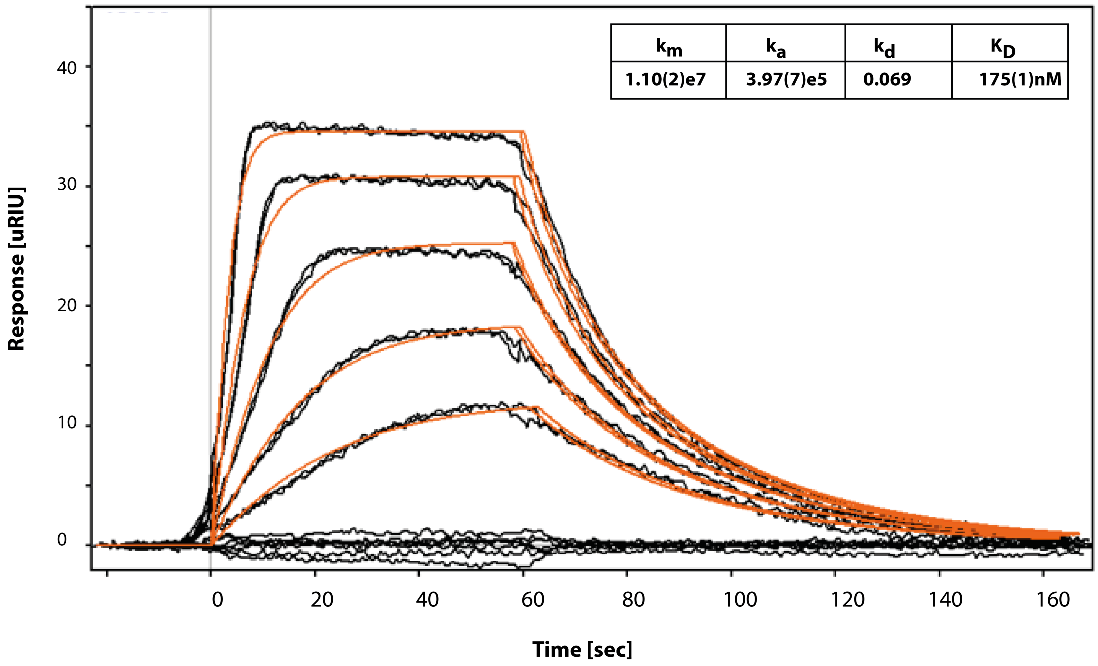

Supplementary Fig. 3. Surface plasmon resonance experiments of Siglec-2 ligands. **a)** Sensorgram of **7** and **b)** of **8** bound to immobilised Siglec-2-Fc. K_D values were obtained by fitting the data to a mass transport limited single binding site model in Scrubber.

Supplementary material:

Synthetic experimental data – Reactions were monitored by thin layer chromatography (TLC) using aluminum plates coated with Silica Gel 60 F254 (Merck). Detection was typically effected under ultraviolet (uv) light where applicable, followed by treatment with H_2SO_4 in EtOH (5% v/v) and charring at ~200 °C. Purification by flash chromatography was achieved by elution through columns of Merck silica gel 60 (0.040-0.063 mm). Waters Sep-Pak Vac C18 Cartridges were used to purify final compounds. ^{1}H and ^{13}C, spectra were recorded using a Bruker Avance 400 MHz (or in some cases 300 MHz) spectrometer. For ^{1}H and ^{13}C spectra, chemical shifts are expressed as parts per million (ppm, δ) and are relative to the solvent used [$CDCl_3$: 7.24 (s) for ^{1}H; 77.0 (t) for ^{13}C; CD_3OD: 3.31 (pent) for ^{1}H; 49.0 (sept) for ^{13}C; D_2O 4.78 (s) for ^{1}H]. Multiplicities are denoted as s (singlet), d (doublet), t (triplet), q (quartet), pent (pentet), dd (doublet of doublets), ddd (doublet of doublet of doublets), dt (doublet of triplets), br (broad), app (apparent) and ABq (AB quartet system). 2D NMR experiments were performed using ^{1}H-^{1}H correlation spectroscopy (COSY) and ^{1}H-^{13}C Heteronuclear Single Quantum Coherence (HSQC) to confirm ^{1}H and ^{13}C assignments. Low-Resolution Mass Spectra (LRMS) were recorded, in electrospray ionisation mode unless otherwise specified, on a Bruker Daltonics esquire 3000 spectrometer, using the positive or negative mode (as indicated). High-Resolution Mass Spectrometry (HRMS) was carried out on an Agilent 1290 HPLC/6530 QTOF with an Jet stream ESI source. All chemicals except

otherwise stated, were purchased from Sigma-Aldrich with the highest purity available. *N*-Acetylneuraminic acid (5-acetamido-3,5-dideoxy-D-*glycero*-D-*galacto*-non-2-ulosonic acid) was obtained from Carbosynth Limited (United Kingdom). The schematic route for the synthesis of **7** and **8** is shown in the **Supplementary Scheme 1**.

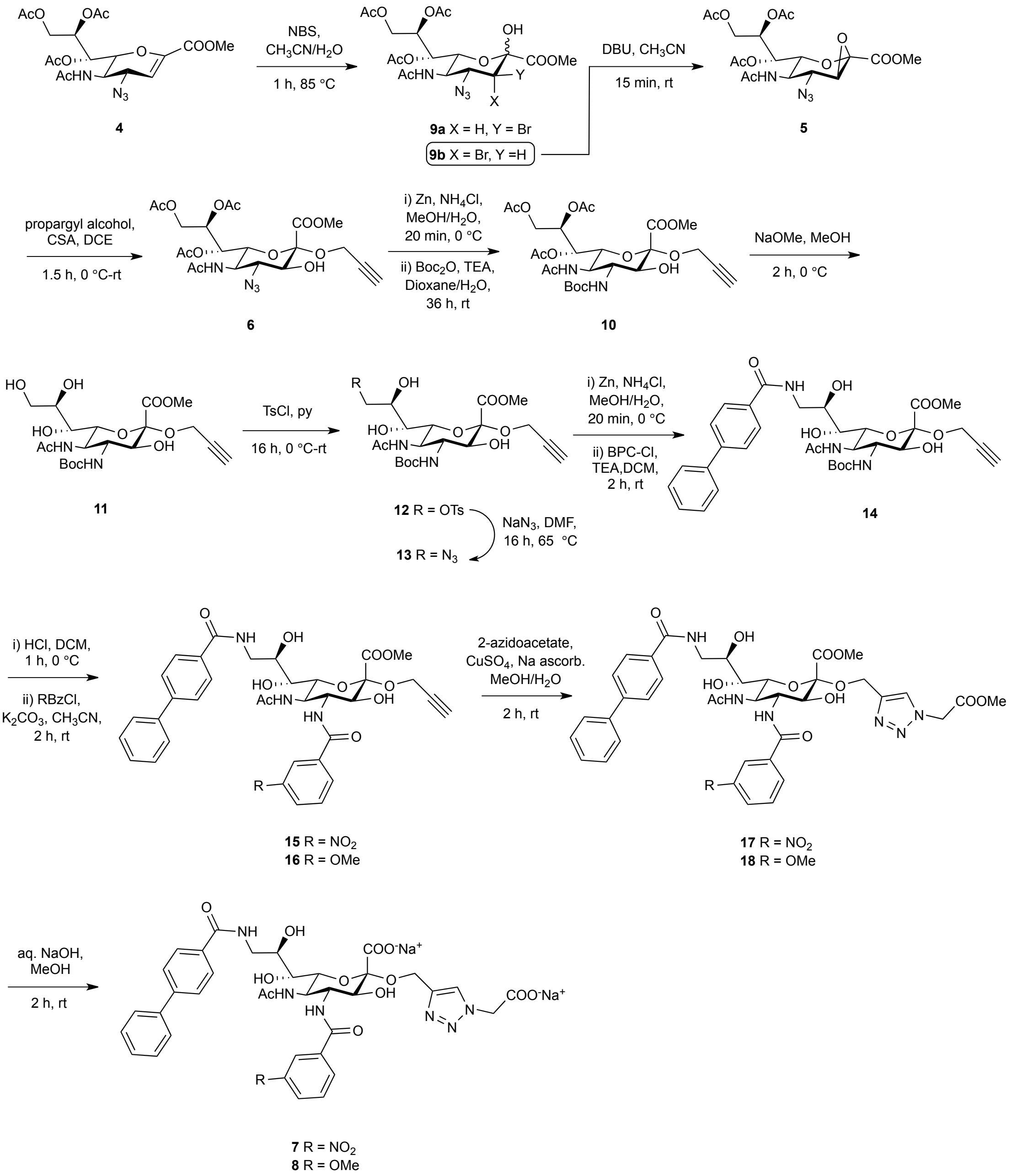

Supplementary Scheme 1. Synthetic route for the generation of **7** and **8**.

Methyl 5-acetamido-7,8,9-tri-*O*-acetyl-2,6-anhydro-4-azido-3,4,5-trideoxy-D-*glycero*-D-*galacto*-non-2-enonate (4) was prepared from commercially available *N*-acetylneuraminic acid over six steps following previously reported procedures.[1-3]

Methyl 5-acetamido-7,8,9-tri-*O*-acetyl-4-azido-3-bromo-3,4,5-trideoxy-β-D-*erythro*-L-*gluco*-non-2-ulopyranosonate (9a) and **methyl 5-acetamido-7,8,9-tri-*O*-acetyl-4-azido-3-bromo-3,4,5-trideoxy-β-D-*erythro*-L-*manno*-non-2-ulopyranosonate (9b)**

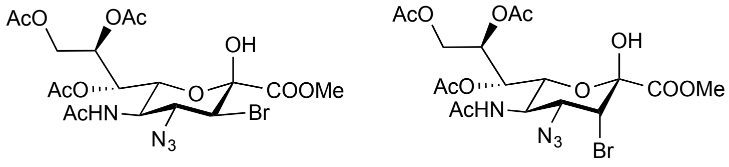

Compound **6** (2.98 g, 6.54 mmol) was dissolved in CH_3CN/H_2O (32/12.8 mL) and NBS (2.80 g, 15.68 mmol) was added once the solution began to reflux. The reaction mixture was stirred for 1 h at 85 °C.[4] The solvent was then evaporated under reduced pressure and the residue was purified by flash silica gel chromatography (toluene/acetone 8:2) to furnish compounds **9a** (0.94 g, 26%) and **9b** (2.36 g, 65%) as white solids.

9a: ^{1}H NMR (300 MHz, CD_3OD): δ 1.91, 1.98, 2.04, 2.08 (4 x s, 12H, NHCOCH_3, $OCOCH_3$ x 3), 3.82-3.89 (m, 1H, H-4), 3.84 (s, 3H, $COOCH_3$), 4.01 (m, 2H, H-5, H-9a), 4.16 (d, J =10.8 Hz, 1H, H-3), 4.37 (m, 2H, H-6, H-9b), 5.08 (m, 1H, H-8), 5.35 (dd, J = 2.4, 6.0 Hz, 1H, H-7); LRMS (ESI): *m/z* [574.9 [$C_{18}H_{25}{}^{79}BrN_4O_{11}+Na]^+$ 95%), [577.0 ($C_{18}H_{25}{}^{81}BrN_4O_{11}+Na)^+$ 100%].

9b: ^{1}H NMR (300 MHz, CD_3OD): δ 1.92, 2.01, 2.03, 2.12 (4 x s, 12H, NHCOCH_3, $OCOCH_3$ x 3), 3.79 (s, 3H, $COOCH_3$), 4.15-4.20 (m, 2H, H-4, H-9a), 4.35 (dd, *J* = 2.1, 10.5 Hz, 1H, H-6), 4.41 (m, 1H, H-5), 4.56 (d, *J*= 3.0 Hz, 1H, H-3), 4.69 (dd, *J* = 2.4, 12.3 Hz, 1H, H-9b), 5.23 (m, 1H, H-8), 5.38 (dd, *J* = 2.4, 6.0 Hz, 1H, H-7); ^{13}C NMR (75.5 MHz, CD_3OD): δ 20.65, 20.81, 20.84 (OCO*C*H_3 x 3), 22.76 (NHCO*C*H_3), 47.30 (C-5), 53.21 (COO*C*H_3), 54.76 (C-3), 61.15 (C-4), 63.76 (C-9), 69.59 (C-7), 72.27 (C-6), 72.84 (C-8), 96.87 (C-2), 169.10 (C-1), 171.84, 171.94, 172.50, 173.50 (NH*C*OCH_3, O*C*OCH_3 x 3); LRMS (ESI): *m/z* [574.9 $(C_{18}H_{25}{}^{79}BrN_4O_{11}+Na)^+$ 95%], [577.0 $(C_{18}H_{25}{}^{81}BrN_4O_{11}+Na)^+$ 100%].

Methyl 5-acetamido-7,8,9-tri-*O*-acetyl-2,3-anhydro-4-azido-4,5-dideoxy-β-D-*erythro*-L-*gluco*-non-2-ulopyranosonate (5)

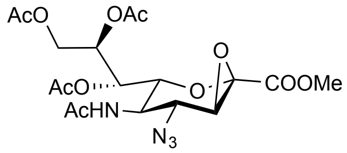

A solution of **9b** (1.901 g, 3.44 mmol) in anhydrous acetonitrile (12 mL) was treated with DBU (0.62 mL, 4.12 mmol) under argon at room temperature.[4] After stirring for 15 min, the reaction mixture was evaporate to a 1/3rd of initial volume and then loaded onto a column of silica-gel column and chromatographed (EtOAc/Hex 2:1) to give compound **5** as a white foam (1.486 g, 91%). ^{1}H NMR (300 MHz, $CDCl_3$): δ 1.96, 2.03, 2.06, 2.11 (4 x s, 12H, NHCOCH_3, $OCOCH_3$ x 3), 3.59 (s, 1H, H-3), 3.83 (s, 3H, $COOCH_3$), 3.86 (m, 1H, H-5), 4.06-4.21 (m, 3H, H-4, H-6, H-9a), 4.50 (dd, *J* = 2.7, 12.3 Hz, 1H, H-9b), 5.24 (m, 1H, H-8), 5.37 (dd, *J* = 3.0, 5.4 Hz, 1H, H-7), 5.58 (br d, *J*= 9.3 Hz, 1H, NHCOCH_3). ^{13}C NMR (75.5 MHz, $CDCl_3$): δ 20.73,

20.78, 20.80 (OCOCH_3 x 3), 23.17 (NHCOCH_3), 46.44 (C-5), 53.45 (COOCH_3), 57.13 (C-3), 59.54 (C-4), 62.28 (C-9), 67.82 (C-7), 70.54 (C-6), 71.11 (C-8), 79.60 (C-2), 164.69 (C-1), 170.31, 170.33, 170.69, 170.80 (NH*C*OCH$_3$, O*C*OCH$_3$ x 3); LRMS (ESI): *m/z* 495.1 [$(M+Na)^+$ 100%].

Methyl (prop-2-ynyl 5-acetamido-7,8,9-tri-*O*-acetyl-4-azido-4,5-dideoxy-D-*glycero*-α-D-*galacto*-non-2-ulopyranosid)onate (6)

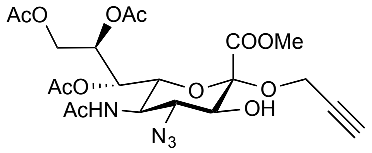

A stirred solution of **5** (1.416 g, 2.99 mmol) in anhydrous DCE (10 mL), at 0 °C under argon, was treated with propargyl alcohol (2.7 mL, 44.85 mmol) and a catalytic amount CSA.[5] After stirring for 15 min the solution was allowed to warm to room temperature and the reaction was continued for 1.5 h. At completion, the solution was concentrated under reduced pressure and the crude residue was diluted with EtOAc and washed with satd. aq. sol. of $NaHCO_3$ and brine. The organic layer was dried over Na_2SO_4, filtered and evaporated to dryness. The residue was purified by flash chromatography on silica gel (EtOAc/Hex 3:1) to give **6** (1.295 g, 82%) as a white solid foam. ^1H NMR (400 MHz, CD_3OD) δ 1.92, 2.00, 2.06, 2.09 (4 x s, 12H, NHCOCH_3, OCOCH$_3$ x 3), 2.83 (t, *J* = 2.5 Hz, 1H, C≡CH), 3.54 (d, *J* = 9.8 Hz, 1H, H-3), 3.70 (dd, *J* = 10.8, 9.7 Hz, 1H, H-4), 3.81 (s, 3H, COOCH$_3$), 3.94 (t, *J* = 10.7 Hz, 1H, H-5), 4.04 (dd, *J* = 12.4, 6.0 Hz, 1H, H-9a), 4.22 (dd, *J* = 12.4, 2.7 Hz, 1H, H-9b), 4.28 – 4.45 (ABq, *J* = 15.4, 2.5 Hz, 2H, OCH_2C≡CH), 4.67 (dd, *J* = 10.7, 2.2 Hz, 1H, H-6), 5.25 (dd, *J* = 9.2, 2.2 Hz, 1H, H-7), 5.38 (ddd, *J* = 8.9, 6.0, 2.7 Hz, 1H,

H-8). ^{13}C NMR (101 MHz, CD_3OD) δ 20.60, 20.88, 20.98 (OCO*C*H_3 x 3), 22.71 (NHCO*C*H_3), 49.15 (C-5), 52.84 (COO*C*H_3), 54.13 (O*C*H_2C≡CH), 63.67 (C-9), 66.49 (C-4), 68.49 (C-7), 69.41 (C-8), 73.80 (C-6), 75.41 (C≡*C*H), 76.35 (C-3), 101.63 (C-2), 170.40, 171.19, 171.72, 172.36, 173.44 (C-1, NH*C*OCH$_3$, O*C*OCH$_3$ x 3). Quaternary *C* of the alkyne was not detected. LRMS (ESI): *m/z* 551.2 [$(M+Na)^+$ 100%].

Methyl (prop-2-ynyl 5-acetamido-7,8,9-tri-*O*-acetyl-4-*tert*-butylcarbamido-4,5-dideoxy-D-*glycero*-α-D-*galacto*-non-2-ulopyranosid)onate (10)

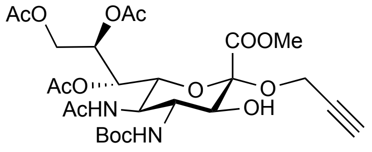

A solution of **6** (1.185 g, 2.24 mmol) in MeOH/H_2O (16:1, 51 mL) was cooled to 0 °C and under vigorous stirring, was treated with NH_4Cl (1.199 g, 22.42 mmol) and Zn dust (1.319 g, 20.18 mmol). After 20 min TLC analysis showed complete consumption of the starting material. The reaction mixture was then filtered on Celite and the solvent was removed under reduced pressure to give the corresponding crude 4-amino derivative which was employed without purification in the next reaction. To a solution of crude 4-amino derivative in a mixture dioxane/H_2O (3:1, 40 mL) were added *tert*-butyldicarbonate (0.590 mL, 2.58 mmol) and triethylamine (0.704 mL, 5.04 mmol) and the reaction was stirrer 16 h at room temperature. The mixture was then concentrated under reduced pressure and purified by silica gel chromatography (EtOAc/Hex 3:1) to give **10** as a white solid (1.039 g, 77% over two steps). ^1H NMR (400 MHz, CD_3OD) δ 1.40 (s, 9H, C(CH_3)$_3$), 1.86, 2.01, 2.07, 2.08 (4 x s, 12H,

$NHCOCH_3$, $OCOCH_3$ x 3), 2.83 (t, *J* = 2.5 Hz, 1H, C≡CH), 3.58 (d, *J* = 9.7 Hz, 1H, H-3), 3.80 (s, 3H, $COOCH_3$), 3.70 (t, *J* = 9.8 Hz, 1H, H-4), 3.94 (t, *J* = 10.7 Hz, 1H, H-5), 4.05 (dd, *J* = 12.4, 6.0 Hz, 1H, H-9a), 4.25 (dd, *J* = 12.4, 2.7 Hz, 1H, H-9b), 4.26 – 4.45 (ABq, *J* = 15.4, 2.5 Hz, 2H, OCH_2C≡CH), 4.67 (dd, *J* = 10.6, 2.2 Hz, 1H, H-6), 5.27 (dd, *J* = 9.0, 2.2 Hz, 1H, H-7), 5.39 (ddd, *J* = 8.8, 6.0, 2.7 Hz, 1H, H-8). ^{13}C NMR (101 MHz, CD_3OD) δ 20.61, 20.87, 21.00 (OCOCH_3 x 3), 22.74 (NHCOCH_3), 28.69 (C(CH_3)$_3$), 49.85 (C-5), 52.76 (COOCH_3), 53.85 (OCH_2C≡CH), 56.22 (C-4), 63.70 (C-9), 68.84 (C-7), 69.57 (C-8), 74.03 (C-6), 75.35 (C≡*C*H), 75.43 (C-3), 80.16 (*C*(CH_3)$_3$), 101.91 (C-3), 158.54, 170.27, 171.31, 171.67, 172.40, 173.43 (C-1, NH*C*O x 2, O*C*OCH_3 x 3). Quaternary *C* of the alkyne was not detected. LRMS (ESI): *m/z* 625.4 [(M+Na)$^+$ 100%].

Methyl (prop-2-ynyl 5-acetamido-4-*tert*-butylcarbamido-4,5-dideoxy-D-*glycero*-α-D-*galacto*-non-2-ulopyranosid)onate (11)

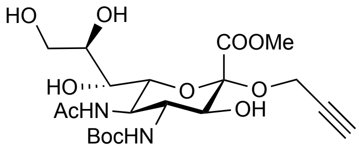

Compound **10** (1.417 mg, 2.35 mmol) was dissolved in anhydrous MeOH (20 mL), cooled to 0 °C and then treated with 1M NaOMe (1.76 mL) under argon. After stirring for 2 h, the reaction mixture was neutralized with Amberlite IR120 H^+ resin, filtered and concentrated under vacuum. Flash chromatography purification (EtOAc/MeOH 9:1) gave **11** as a white solid (1.019 mg, 91%). ^{1}H NMR (400 MHz, CD_3OD) δ 1.44 (s, 9H, C(CH_3)$_3$), 1.96 (s, 3H, $NHCOCH_3$), 2.82 (t, *J* = 2.5 Hz, 1H, C≡CH), 3.45 (dd, *J* = 9.0, 1.4 Hz, 1H, H-7), 3.58 – 3.68 (m, 2H, H3, H-9a), 3.73 –

3.81 (m, 2H, H-8, H-9b), 3.82 (s, 3H, $COOCH_3$), 3.97 (dd, J = 10.4, 8.4 Hz, 1H, H-4), 3, 4.09 (t, J = 10.4 Hz, 1H, H-5), 4.30 (dd, J = 10.7, 1.4 Hz, 1H, H-6), 4.38 – 4.42 (ABq, J = 2.5 Hz, 2H, $OC\mathit{H}_2C{\equiv}CH$). ^{13}C NMR (101 MHz, CD_3OD) δ 22.64 ($NHCOC\mathit{H}_3$), 28.71 ($C(\mathit{C}H_3)_3$), 50.89 (C-5), 52.76 ($COO\mathit{C}H_3$), 53.01 ($O\mathit{C}H_2C{\equiv}CH$), 56.09 (C-4), 65.12 (C-9), 70.36 (C-7), 71.64 (C-8), 75.00 (C-3, C-6), 75.38 ($OCH_2C{\equiv}\mathit{C}H$), 80.33 ($\mathit{C}(CH_3)_3$), 101.42 (C-2), 158.74 (C-1), 170.07, 174.45 (NH*C*O x 2). Quaternary *C* of the alkyne was not detected. LRMS (ESI): *m/z* 499.2 [$(M+Na)^+$ 100%].

Methyl (prop-2-ynyl 5-acetamido-9-azido-4-*tert*-butylcarbamido-4,5,9-trideoxy-D-*glycero*-α-D-*galacto*-non-2-ulopyranosid)onate (13)

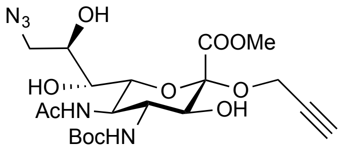

To a solution of **11** (846 mg, 1.78 mmol) in anhydrous pyridine (16 mL) was added *p*-toluenesulfonyl chloride (406 mg, 2.13 mmol) at 0 °C. The reaction mixture was stirred under argon for 2 h and allowed to warm to room temperature and stirred for a further 16 h.[6] TLC confirmed consumption of starting material and the reaction was quenched with MeOH (10 mL) and stirred at room temeprature for 15 min. The solvent was removed under reduced pressure and stripped with toluene as needed. Silica gel chromatography (3:1 EtOAc/Hex) yielded the title compound **12** (909 mg, 81%) which was immediately employed for the subsequent S_N2 reaction. LRMS (ESI): *m/z* 654.2 [$(M+Na)^+$ 100%].

Compound **12** (817 mg, 1.29 mmol) was dissolved in anhydrous DMF (20 mL) followed by sequential addition of NaN_3 (419 mg, 6.45 mmol) and 18-crown-6 (170 mg, 0.64 mmol) under an argon atmosphere.[6] The reaction was heated to 65 °C and stirred for 24 h before being concentrated under reduced pressure. The residue was dissolved in EtOAc, washed with H_2O, brine and the organic phase was dried over Na_2SO_4. The solvent was removed under vacuum and the residue was purified by flash silica gel chromatography (EtOAc/Hex 5:1) to give the desired compound **13** as a white solid.). ^{1}H NMR (400 MHz, CD_3OD) δ 1.44 (s, 9H, $C(CH_3)_3$), 1.97 (s, 3H, NHCOC*H₃*), 2.82 (t, *J* = 2.5 Hz, 1H, C≡CH), 3.36 (dd, *J* = 12.8, 6.3 Hz, 1H, H-9a), 3.41 (dd, *J* = 9.3, 1.4 Hz, 1H, H-7), 3.51 (dd, *J* = 12.8, 2.6 Hz, 1H, H-9b), 3.66 (d, *J* = 8.3 Hz, 1H, H-3), 3.82 (s, 3H, $COOCH_3$), 3.91 (ddd, *J* = 9.1, 6.3, 2.6 Hz, 1H, H-8), 3.97 (dd, *J* = 10.4, 8.2 Hz, 1H, H-4), 4.08 (t, *J* = 10.6 Hz, 1H, H-5), 4.31 (dd, *J* = 10.7, 1.4 Hz, 1H, H-6), 4.40 – 4.49 (ABq, *J* = 2.5 Hz, 2H, OCH_2C≡CH). ^{13}C NMR (101 MHz, CD_3OD) δ 22.63 (NHCOCH_3), 28.71 (C(CH_3)$_3$), 50.85 (C-5), 52.79 (COOCH_3), 53.02 (OCH_2C≡CH), 55.88 (C-9), 56.09 (C-4), 70.75 (C-7), 70.94 (C-8), 74.73 (C-6), 74.95 (C-3), 75.42 (OCH_2C≡*C*H), 80.35 (*C*(CH_3)$_3$), 101.46 (C-2), 158.70 (C-1), 170.02, 174.51, (NH*C*O x 2). Quaternary *C* of the alkyne was not detected. LRMS (ESI): *m/z* 524.3 [$(M+Na)^+$ 100%].

Methyl (prop-2-ynyl 5-acetamido-9-biphenylamido-4-*tert*-butylcarbamido-4,5,9-trideoxy-D-*glycero*-α-D-*galacto*-non-2-ulopyranosid)onate (14)

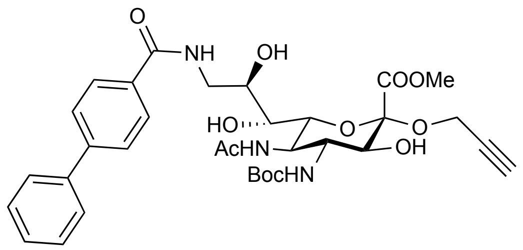

Compound **13** (364 mg, 0.723 mmol) was dissolved in a mixture of MeOH/H_2O (16:1, 17 mL). The reaction was cooled to 0 °C and under vigorous stirring, NH_4Cl (387 mg, 7.23 mmol) and Zn dust (424 mg, 6.507 mmol) were added.[6] After 20 min the solution was filtered through Celite and the solvent was removed under vacuum. The crude residue was taken up in anhydrous DCM (12 mL) to which at 0 °C under argon was added 4-biphenylcarbonyl chloride (243 mg, 1.085 mmol) and triethylamine (0.504 mL, 3.615 mmol).[6] After 10 min the solution was let to warm to room temperature with stirring continuing for 2 h. The reaction mixture was then diluted with DCM and washed with 1 M aq. HCl. The aqueous layer was extracted two times with DCM. The combined organic layers were then successively washed with satd. aq. sol. $NaHCO_3$ and brine before being dried over Na_2SO_4 and then concentrated to dryness under reduced pressure. Purification by flash silica gel chromatography (EtOAc → EtOAc/acetone 9:1) gave the title compound **14** as a white foam (327 mg, 69% over 2 steps). ^{1}H NMR (400 MHz, CD_3OD) δ 1.44 (s, 9H, $C(CH_3)_3$), 1.93 (s, 3H, NHCOC*H*$_3$), 2.81 (t, *J* = 2.5 Hz, 1H, C≡CH), 3.41 (dd, *J* = 8.9, 1.4 Hz, 1H, H-7), 3.53 (dd, *J* = 13.9, 7.1 Hz, 1H, H-9a), 3.69 (d, *J* = 8.4 Hz, 1H, H-3), 3.78 – 3.85 (m, 1H, H-9b), 3.82 (s, 3H, $COOCH_3$), 3.93 – 4.03 (m, 2H, H-4, H-8), 4.14 (t, *J* = 10.7 Hz, 1H, H-5), 4.37 (dd, *J* = 10.7, 1.4 Hz, 1H, H-6), 4.42 – 4.53 (ABq, *J* = 15.4, 2.5 Hz, 2H, OC*H*$_2$C≡CH), 7.25 – 7.58 (m, 3H, Ar*H*), 7.58 – 7.81 (m, 4H, Ar*H*), 7.84 – 8.05 (m, 2H, Ar*H*). ^{13}C NMR (101 MHz, CD_3OD) δ 22.67 (NHCOC*H*$_3$), 28.72 (C(*C*H$_3$)$_3$), 45.29 (C-9), 50.83 (C-5), 52.78 (COO*C*H$_3$), 53.04

(OCH$_2$C≡CH), 56.16 (C-4), 70.51 (C-8), 71.96 (C-7), 74.83 (C-3),74.94 (C-6), 75.43 (OCH$_2$C≡CH), 80.32 (C(CH$_3$)$_3$), 101.45 (C-2), 128.01, 128.10, 128.95, 129.28, 130.01, 134.27, 141.24, 145.67 (ArC x 8), 158.69 (C-1), 170.03, 170.61, 174.27 (NHCO x 3). Quaternary C of the alkyne was not detected. LRMS (ESI): *m/z* 678.4 [$(M+Na)^+$ 100%].

Methyl [prop-2-ynyl 5-acetamido-9-biphenylamido-4-(3'-nitrobenzamido)-4,5,9-trideoxy-D-*glycero*-α-D-*galacto*-non-2-ulopyranosid]onate (15)

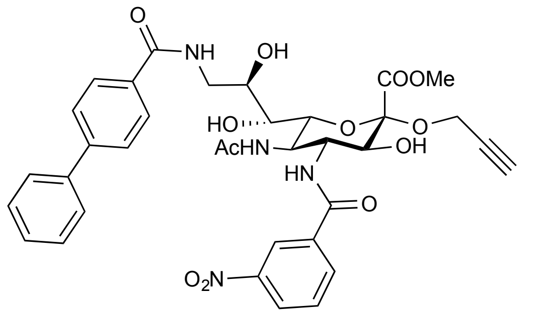

To an ice-cooled solution of **14** (311 mg, 0.474 mmol) in DCM (15 mL), HCl (32 %, 10 drops) was added.[6] After stirring for 1 h, TLC showed complete conversion into the desired amine. The reaction mixture was diluted with acetonitrile (15 mL) and the chlorinated solvent was removed under reduced pressure. The resulting mixture was the treated with 1 M aq. K_2CO_3 (9.48 mL) and 3-nitrobenzoyl chloride (134 mg, 0.711 mmol) and stirred for 1.5 h at room temperature. After the addition of water, CH_3CN was evaporated under reduced pressure and the product was extracted with EtOAc (3 times), washed with brine and dried over Na_2SO_4 and concentrated. The residue was purified by silica gel chromatography (EtOAc/MeOH 96:4) to give **15** as an off white solid (257 mg, 77% over two steps). ^{1}H NMR (400 MHz, CD$_3$OD) δ 1.86 (s, 3H, NHCOCH_3), 2.84 (t, J = 2.5 Hz, 1H, C≡CH), 3.47 (dd, J = 9.0, 1.4 Hz, 1H, H-

7), 3.56 (dd, J = 13.9, 7.1 Hz, 1H, H-9a), 3.85 (d, J = 9.0 Hz, 1H, H-9b), 3.85 (s, 3H, $COOCH_3$), 3.91 (d, J = 9.0 Hz, 1H, H-3), 4.00 (ddd, J = 9.0, 7.1, 3.2 Hz, 1H, H-8), 4.39 – 4.59 (m, 4H, H-5, H-6, OCH_2C≡CH), 4.63 (dd, J = 10.1, 9.0 Hz, 1H, H-4), 7.32 – 7.56 (m, 3H, Ar*H*), 7.56 – 7.82 (m, 6H, Ar*H*), 7.86 – 8.02 (m, 2H, Ar*H*), 8.20 (dt, J = 7.8, 1.3 Hz, 1H, Ar*H*), 8.38 (ddd, J = 8.2, 2.3, 1.1 Hz, 1H, Ar*H*), 8.68 (t, J = 2.0 Hz, 1H, Ar*H*). ^{13}C NMR (101 MHz, CD_3OD) δ 22.63 (NHCOCH_3), 45.26 (C-9), 50.26 (C-5), 52.87 (COO*C*H_3), 53.32 (O*C*H_2C≡CH), 55.84 (C-4), 70.68 (C-8), 71.76 (C-7), 74.46 (C-3), 74.72 (C-6), 75.55 (C≡*C*H), 101.55 (C-2), 123.45, 127.02, 128.02, 128.10, 128.97, 129.08, 130.01, 130.99, 134.22, 134.44, 137.53, 141.22, 145.70, 149.65 (Ar*C* x 14), 168.45, 170.01, 170.70, 174.08 (C-1, NH*C*O x 3). Quaternary *C* of the alkyne was not detected. LRMS (ESI): *m/z* 727.4 [(M+Na)$^+$ 100%].

Methyl [prop-2-ynyl 5-acetamido-9-biphenylamido-4-(3'-methoxybenzamido)-4,5,9-trideoxy-D-*glycero*-α-D-*galacto*-non-2-ulopyranosid]onate (16)

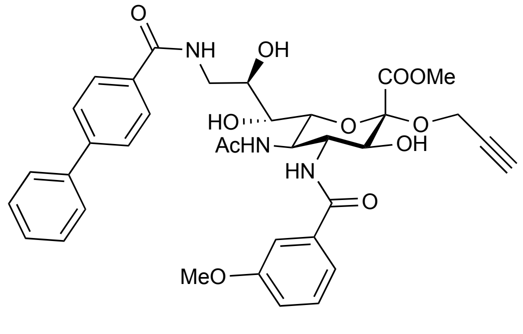

According to the procedure described for **15**, compound **14** (292 mg, 0.445 mmol) was treated with HCl in DCM to give the corresponding 4-amino derivative which upon treatment with 3-methoxybenzoyl chloride (93 μL, 0.682 mmol) and 1 M aq. K_2CO_3 (8.9 mL) and subsequent purification (EtOAc/MeOH 95:5) gave the title compound **16** (239 mg, 78%).^{1}H NMR (400 MHz, CD_3OD) δ 1.87 (s, 3H,

NHCOCH_3), 2.84 (t, J = 2.5 Hz, 1H, C≡CH), 3.46 (dd, J = 9.0, 1.3 Hz, 1H, H-7), 3.55 (dd, J = 13.9, 7.1 Hz, 1H, H-9a), 3.82 (m, 1H, H-9b), 3.84 (s, 3H, ArOCH$_3$), 3.85 (s, 3H, COOCH$_3$), 3.89 (d, J = 9.0 Hz, 1H, H-3), 3.99 (ddd, J = 9.0, 7.0, 3.1 Hz, 1H, H-8), 4.35 – 4.66 (m, 5H, H-4, H-5, H-6, OCH_2C≡CH), 7.01 – 7.16 (m, 1H, Ar*H*), 7.31 – 7.52 (m, 6H, Ar*H*), 7.62 – 7.81 (m, 4H, Ar*H*), 7.93 (m, 2H, Ar*H*). ^{13}C NMR (101 MHz, CD$_3$OD) δ 22.63 (NHCOCH_3), 45.26 (C-9), 50.34 (C-5), 52.86 (COO*C*H$_3$), 53.26 (O*C*H$_2$C≡CH), 55.61 (C-4), 55.89 (ArO*C*H$_3$), 70.65 (C-8), 71.81 (C-7), 74.59 (C-3), 74.76 (C-6), 75.53 (C≡*C*H), 101.58 (C-2), 113.66, 118.54, 120.57, 128.03, 128.10, 128.97, 129.08, 130.01, 130.60, 134.25, 137.18, 141.24, 145.71, 161.20 (Ar*C* x 14), 170.04, 170.68, 170.95, 174.13 (C-1, NH*C*O x 3). Quaternary *C* of the alkyne was not detected. LRMS (ESI): *m/z* 712.5 [(M+Na)$^+$ 100%].

Methyl [(1'-methylacetate-[1',2',3']-triazol-4'-yl)methyl 5-acetamido-9-biphenylamido-4-(3'-nitrobenzamido)-4,5,9-trideoxy-D-*glycero*-α-D-*galacto*-non-2-ulopyranosid]onate (17)

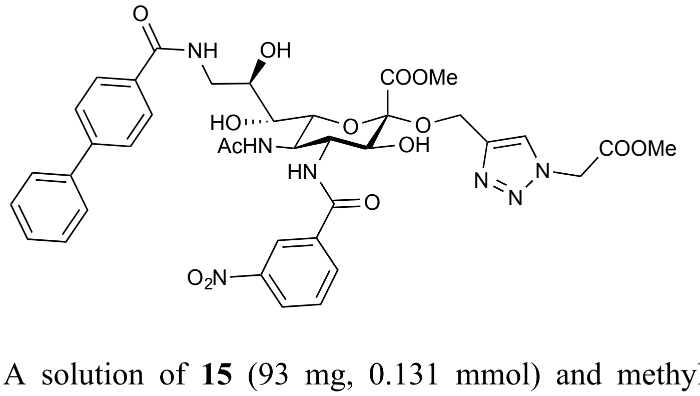

A solution of **15** (93 mg, 0.131 mmol) and methyl 2-azidoacetate (38 μL, 0.393 mmol) in methanol (3.5 mL) was treated with a 0.1 M aq. sol. sodium ascorbate (0.65 mL) and 0.1 M aq. sol. CuSO$_4$•5H$_2$O (0.65 mL). The resulting mixture was stirred for 2 h at room temperature before being concentrated. The crude residue was dissolved

in EtOAc, washed with satd. aq. sol NH_4Cl, brine, dried over Na_2SO_4 and concentrated under reduced pressure. Purification by silica gel chromatography (MeOH 5% → 10% in EtOAc) gave the title compound **17** as a white solid (90 mg, 84%). ^{1}H NMR (400 MHz, CD_3OD) δ 1.87 (s, 3H, NHCOC*H₃*), 3.51 (app d, *J* = 8.8 Hz, 1H, H-7), 3.56 (dd, *J* = 13.8, 7.1 Hz, 1H, H-9a), 3.76 (s, 3H, , CH_2COOCH_3), 3.85 (s, 3H, $COOCH_3$), 3.86 (m, 1H, H-9b), 3.94 (d, *J* = 9.3 Hz, 1H, H-3), 4.01 (ddd, *J* = 9.4, 7.2, 3.1 Hz, 1H, H-8), 4.48 (t, *J* = 10.5 Hz, 1H, H-5), 4.61 – 4.74 (m, 2H, H-4, H-6), 4.88 – 5.07 (ABq, *J* = 12.6 Hz, 2H, OCH_2C), 5.32 (s, 2H, CH_2COOCH_3), 7.32 – 7.41 (m, 1H, Ar*H*), 7.45 (dd, *J* = 8.4, 6.7 Hz, 2H, Ar*H*), 7.59 – 7.75 (m, 5H, Ar*H*), 7.87 – 7.95 (m, 2H, Ar*H*), 8.13 (s, 1H, triazole CH), 8.17 (dt, *J* = 7.8, 1.3 Hz, 1H, Ar*H*), 8.36 (ddd, *J* = 8.2, 2.3, 1.0 Hz, 1H, Ar*H*), 8.65 (t, *J* = 2.0 Hz, 1H, Ar*H*). ^{13}C NMR (101 MHz, CD_3OD) δ 22.66 (NHCOCH_3), 45.40 (C-9), 50.44 (C-5), 51.60 (CH_2COOCH_3), 52.91 (COOCH_3), 53.32 (CH_2COOCH_3), 55.67 (C-4), 58.92 (OCH_2C), 70.71 (C-8), 71.99 (C-7), 74.55 (C-3), 74.89 (C-6), 102.20 (C-2), 127.18 (triazole *C*H), 123.42, 126.99, 128.00, 128.09, 128.96, 129.08, 130.01, 131.00, 134.24, 134.41, 137.53, 141.19, 145.64, 149.62 (Ar*C* x 14), 168.46, 168.87, 170.25, 170.59, 174.10 (C-1, CH_2COOCH_3, NH*C*O x 3). Quaternary *C* of the triazole was not detected. LRMS (ESI): *m/z* 842.4 [$(M+Na)^+$ 100%].

Methyl [(1'-methylacetate-[1',2',3']-triazol-4'-yl)methyl 5-acetamido-9-biphenylamido-4-(3'-methoxybenzamido)-4,5,9-trideoxy-D-*glycero*-α-D-*galacto*-non-2-ulopyranosid]onate (18)

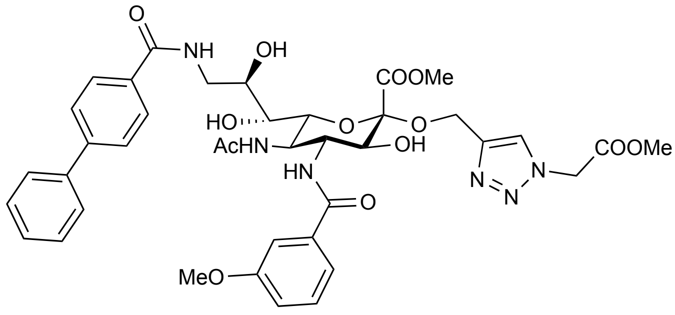

According to the procedure described for **17**, compound **16** (88 mg, 0.128 mmol) was treated with methyl 2-azidoacetate (37 μL, 0.384 mmol), 0.1 M aq. sol. sodium ascorbate (0.64 mL) and 0.1 M aq. sol. $CuSO_4$•$5H_2O$ (0.64 mL). Purification of the reaction mixture yielded derivative **18** as a white solid (83 mg, 81%). ^{1}H NMR (400 MHz, CD_3OD) δ 1.87 (s, 3H, NHCOC$\mathit{H_3}$), 3.49 (dd, *J* = 9.0, 1.3 Hz, 1H, H-7), 3.55 (dd, *J* = 13.9, 7.1 Hz, 1H, H-9a), 3.77 (s, 3H, , CH_2COOC$\mathit{H_3}$), 3.81 (m, 1H, H-9b), 3.83 (s, 3H, ArOCH_3), 3.85 (s, 3H, $COOCH_3$), 3.89 (d, *J* = 9.3 Hz, 1H, H-3), 4.00 (ddd, *J* = 9.1, 7.1, 3.2 Hz, 1H, H-8), 4.43 (t, *J* = 10.6 Hz, 1H, H-5), 4.55 – 4.72 (m, 2H, H-4, H-6), 4.87 – 5.07 (ABq, *J* = 12.5 Hz, 2H, OC$\mathit{H_2}$C), 5.32 (s, 2H, C$\mathit{H_2}$$COOCH_3$), 6.99 – 7.13 (m, 1H, Ar*H*), 7.30 – 7.41 (m, 4H, Ar*H*), 7.43 – 7.50 (m, 2H, Ar*H*), 7.62 – 7.76 (m, 4H, Ar*H*), 7.86 – 7.96 (m, 2H, Ar*H*), 8.12 (s, 1H, triazole CH). ^{13}C NMR (101 MHz, CD_3OD) δ 22.64 (NHCOC$\mathit{H_3}$), 45.39 (C-9), 50.50 (C-5), 51.58 (*C*$H_2$$COOCH_3$), 52.87 (COO*C*$H_3$), 53.31 ($CH_2$COO*C*$H_3$*)*, 55.40 (C-4), 55.88 (ArO*C*H_3), 58.88 (O*C*H_2C), 70.67 (C-8), 72.03 (C-7), 74.67 (C-3), 74.94 (C-6), 102.25 (C-2), 127.11 (triazole *C*H), 118.51, 120.54, 128.11, 128.97, 129.09, 130.02, 130.61, 134.28, 137.21, 141.24, 145.68, 146.52, 161.19, (Ar*C* x 13), 168.87, 170.28, 170.61, 170.99, 174.14 (C-1, CH_2*C*$OOCH_3$, NH*C*O x 3). Quaternary *C* of the triazole was not detected. x LRMS (ESI): *m/z* 827.6 [$(M+Na)^+$ 100%].

[(1'-carboxymethyl-[1',2',3']-triazol-4'-yl)methyl 5-acetamido-9-biphenylamido-4-(3'-nitrobenzamido)-4,5,9-trideoxy-D-*glycero*-α-D-*galacto*-non-2-ulopyranosidonic acid, disodium salt (7)

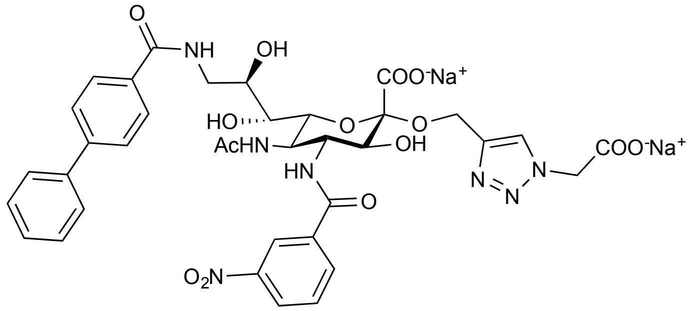

A solution of **17** (88 mg, 0.107 mmol) in methanol (3 mL) was treated with 1 M NaOH (2 mL) at 0 °C for 15 min, and stirred for 1.5-2 h at room temperature. The reaction mixture was then acidified with Amberlite IR120 H^+ resin, filtered and concentrated *in vacuo*. The crude residues were dissolved in water and the pH was adjusted to ~7.2 with dilute NaOH to form the sodium salt. Purification was carried out using a Sep-Pak-12cc C18 Cartridge and the fractions containing the desired compound were lyophilized to give **7** (81 mg, 89%). ^{1}H NMR (400 MHz, D_2O) δ 1.89 (s, 3H, NHCOCH_3), 3.59 – 3.65 (m, 2H, H-7, H-9a), 3.81 – 3.86 (m, 2H, H-9b, H-3), 4.08 – 4.13 (m, 2H, H-6, H-8), 4.30 – 4.38 (m, 2H, H-4, H-5), 4.75 (m, 1H, OCH_2C), 4.98 – 5.02 (m, 1H, OCH_2C), 5.01 (s, 1H, CH_2COONa) 7.44 – 7.54 (m, 3H, Ar*H*), 7.68 – 7.77 (m, 5H, Ar*H*), 7.84 (d, *J* = 8.1 Hz, 2H, Ar*H*), 7.95 (s, 1H, triazole CH), 8.11 (d, *J* = 7.9 Hz, 1H, Ar*H*), 8.43 (d, *J* = 7.9 Hz, 1H, Ar*H*), 8.58 (s, 1H, Ar*H*).^{13}C NMR (101 MHz, D_2O) δ 21.79 (NHCOCH_3), 42.79 (C-9), 49.20 (C-5), 53.03 (CH_2COONa*)*, 56.18 (C-4) 57.60 (O*C*H_2C), 69.76 (C-7), 70.05 (C-8), 72.78 (C-6), 73.48 (C-3), 98.90 (C-2), 122.36, 126.04, 126.64, 127.02, 127.74, 128.31, 129.12, 130.21, 132.28, 133.42, 134.98, 139.34, 143.77, 147.88 (Ar*C* x 14, triazole *C*H),

169.31, 170.67, 172.40, 173.09, 174 (C-1, CH_2*C*OONa, NH*C*O x 3). Quaternary *C* of the triazole was not detected. LRMS (ESI): *m/z* 790.7 [$(M-2Na)^-$ 100%]. HRMS Calcd for $C_{36}H_{37}N_7O_{14}$ $[M-2Na]^-$: 790.2326, found 790.2312.

[(1'-carboxymethyl-[1',2',3']-triazol-4'-yl)methyl 5-acetamido-9-biphenylamido-4-(3'-methoxybenzamido)-4,5,9-trideoxy-D-*glycero*-α-D-*galacto*-non-2-ulopyranosidonic acid, disodium salt (8)

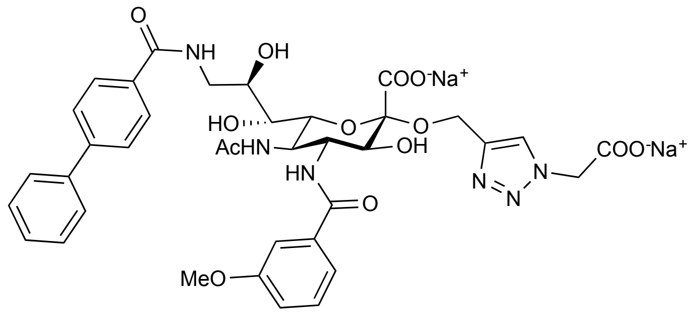

According to the procedure described for **7**, compound **18** (49 mg 0.061 mmol), was deprotected and purified to give **8** (45 mg, 91%). ^{1}H NMR (400 MHz, D_2O) δ 1.88 (s, 3H, NHCOC*H$_3$*), 3.60 – 3.65 (m, 2H, H-7, H-9a), 3.79 – 3.86 (m, 2H, H-9b, H-3), 3.89 (s, 1H, OCH_3), 4.06 – 4.12 (m, 2H, H-6, H-8), 4.27 – 4.35 (m, 2H, H-4, H-5), 4.76 (m, 1H, OC*H$_2$*C), 4.98 – 5.02 (m, 1H, OC*H$_2$*C), 5.01 (s, 1H, CH_2COONa), 7.21 (ddd, *J* = 8.3, 2.7, 1.0 Hz, 1H, Ar*H*), 7.30 – 7.35 (m, 2H, Ar*H*), 7.45 – 7.57 (m, 4H, Ar*H*), 7.72 – 7.79 (m, 4H, Ar*H*), 7.86 – 7.89 (m, 2H, Ar*H*), 7.97 (s, 1H, triazole CH).^{13}C NMR (101 MHz, D_2O) δ 21.79 (NHCOC*H$_3$*), 42.78 (C-9), 49.24 (C-5), 53.04 (*CH$_2$*COONa*)*, 55.49 (OCH_3), 55.90 (C-4) 57.61 (O*C*H_2C), 69.76 (C-7), 70.01 (C-8), 72.82 (C-6), 73.49 (C-3), 98.95 (C-2), 112.35, 118.15, 119.76, 126.07, 127.07,

127.77, 128.32, 129.14, 130.14, 132.36, 135.08, 139.42, 144.02, 158.98 (Ar*C* x 14, triazole *C*H), 170.78, 171.35, 172.43, 173.10, 174.04 (C-1, CH_2*C*OONa, NH*C*O x 3). Quaternary *C* of the triazole was not detected. LRMS (ESI): *m/z* 775.6 [$(M-2Na)^-$ 100%]. HRMS Calcd for $C_{37}H_{40}N_6O_{13}$ $[M-2Na]^-$: 775.2581, found 775.2551.

NMR SPECTRA:

Methyl 5-acetamido-7,8,9-tri-*O*-acetyl-4-azido-3-bromo-3,4,5-trideoxy-β-D-*erythro*-L-*manno*-non-2-ulopyranosonate (**9b**)

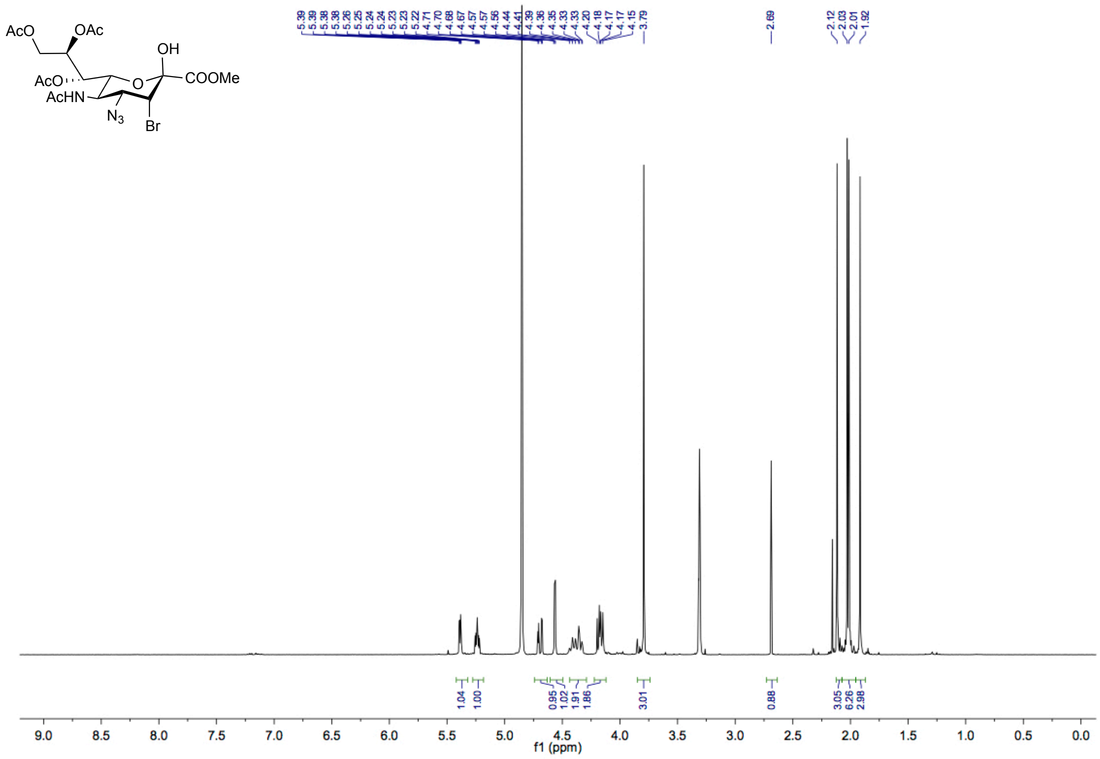

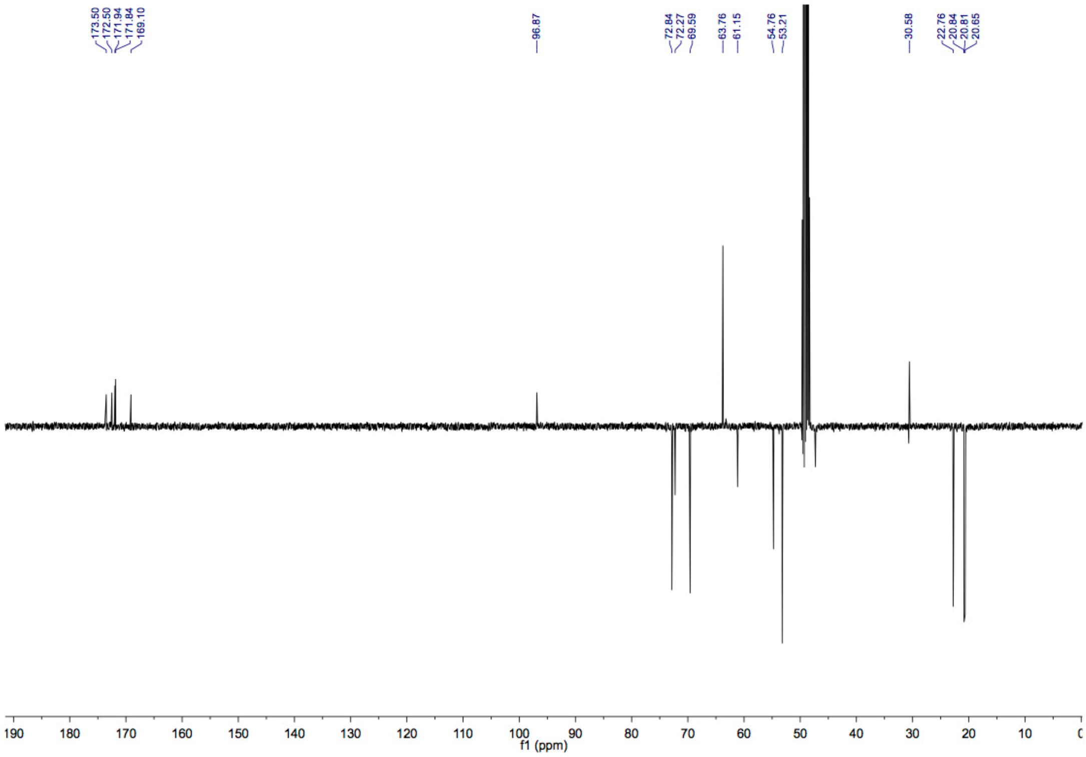

Methyl 5-acetamido-7,8,9-tri-*O*-acetyl-2,3-anhydro-4-azido-4,5-dideoxy-β-D-*erythro*-L-*gluco*-non-2-ulopyranosonate (**5**)

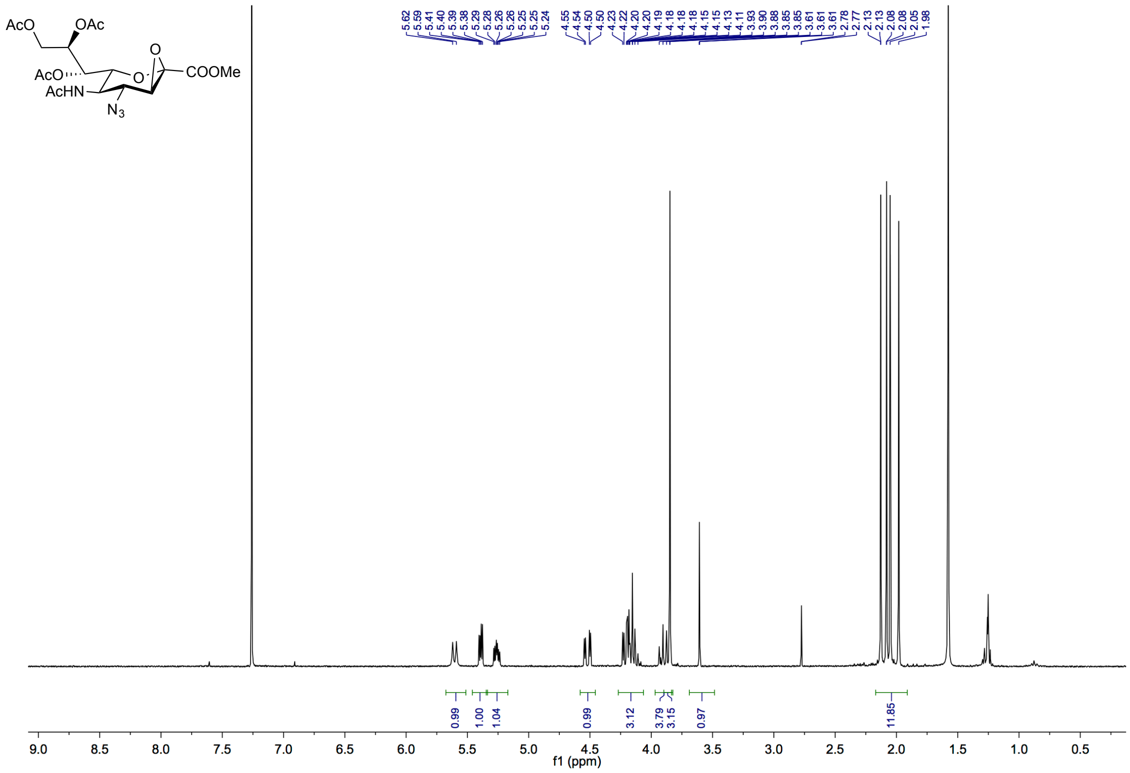

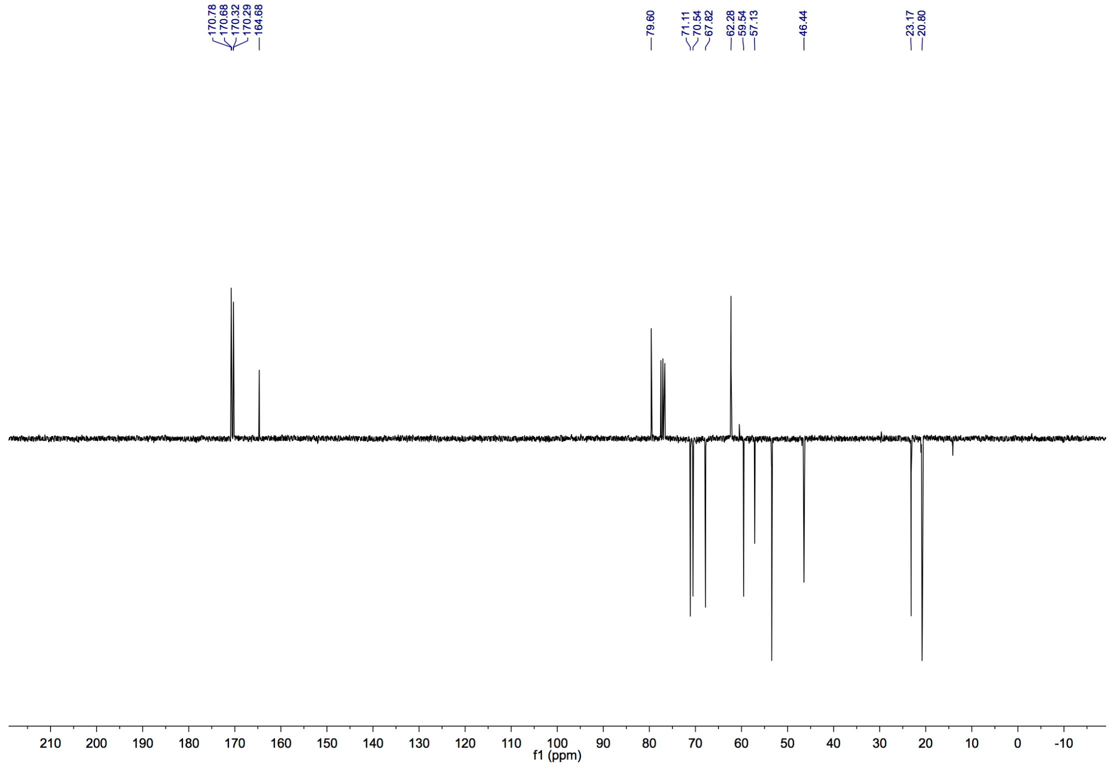

Methyl (prop-2-ynyl 5-acetamido-7,8,9-tri-*O*-acetyl-4-azido-4,5-dideoxy-D-*glycero*-α-D-*galacto*-non-2-ulopyranosid)onate (**6**)

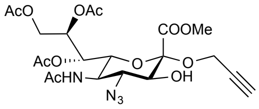

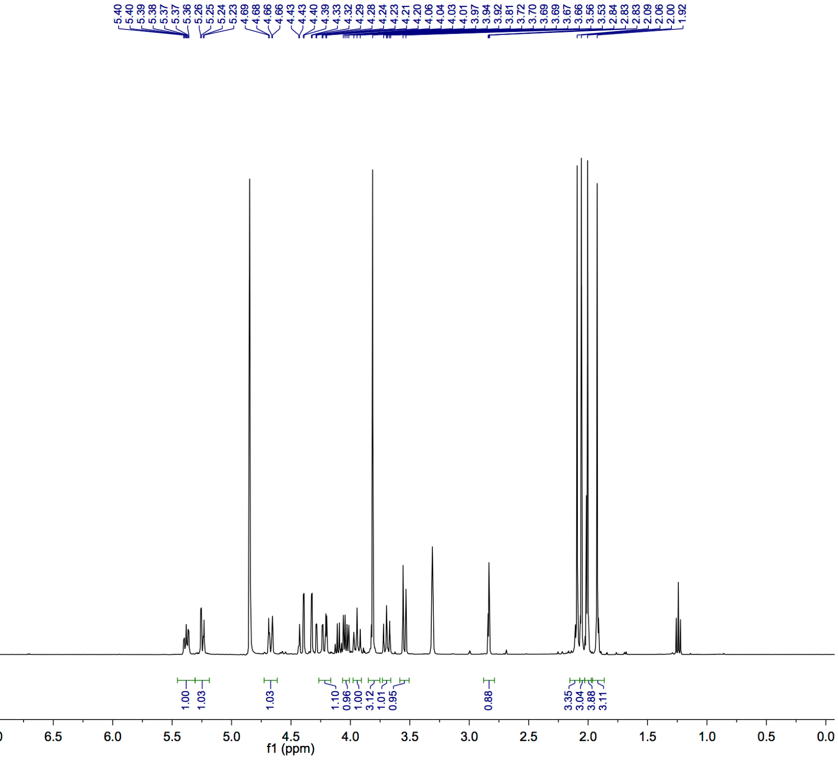

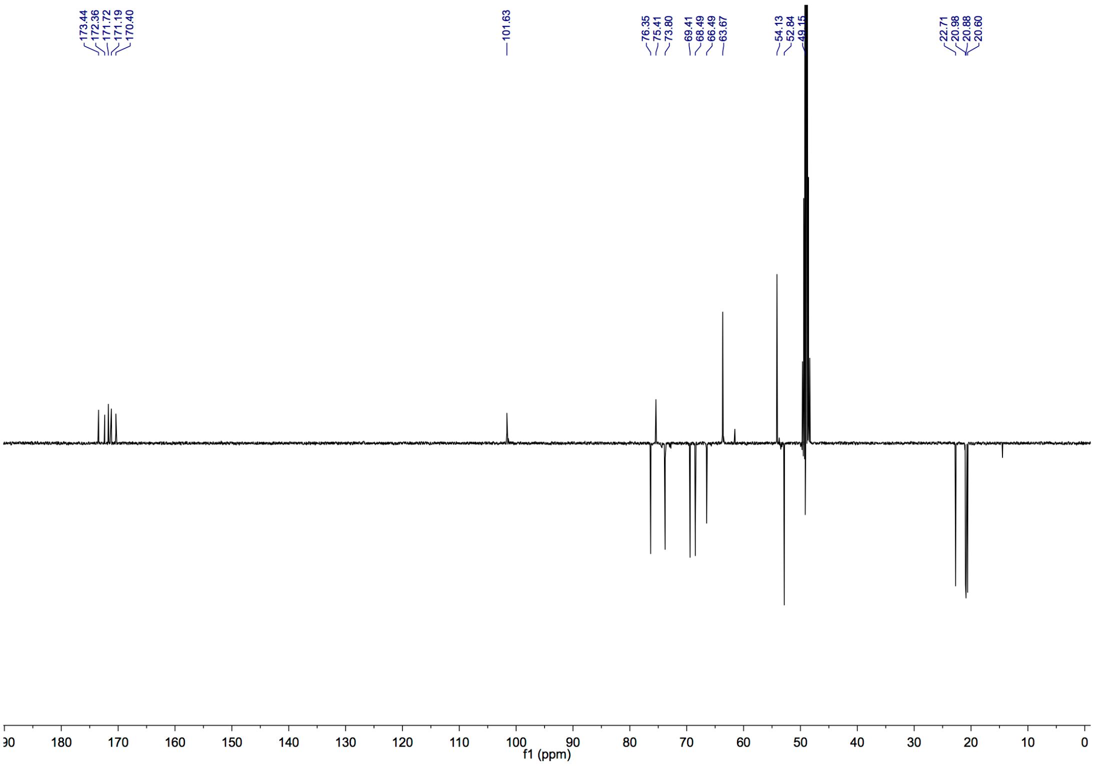

Methyl (prop-2-ynyl 5-acetamido-7,8,9-tri-*O*-acetyl-4-*tert*-butylcarbamido-4,5-dideoxy-D-*glycero*-α-D-*galacto*-non-2-ulopyranosid)onate (**10**)

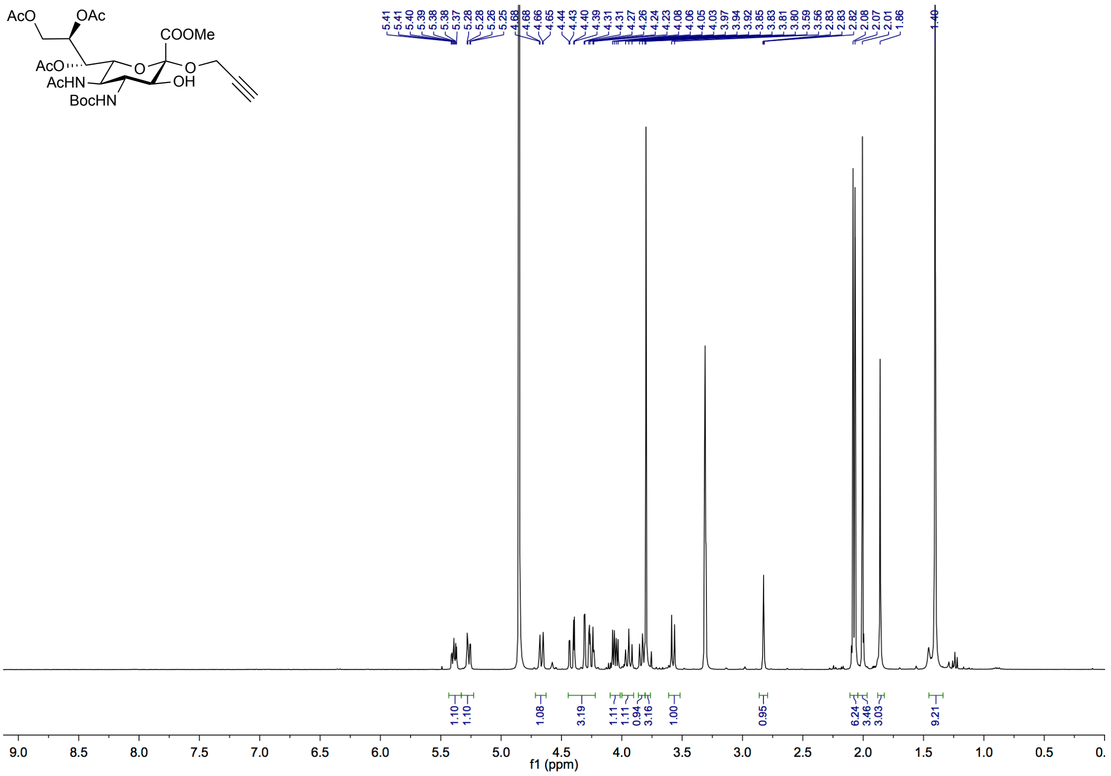

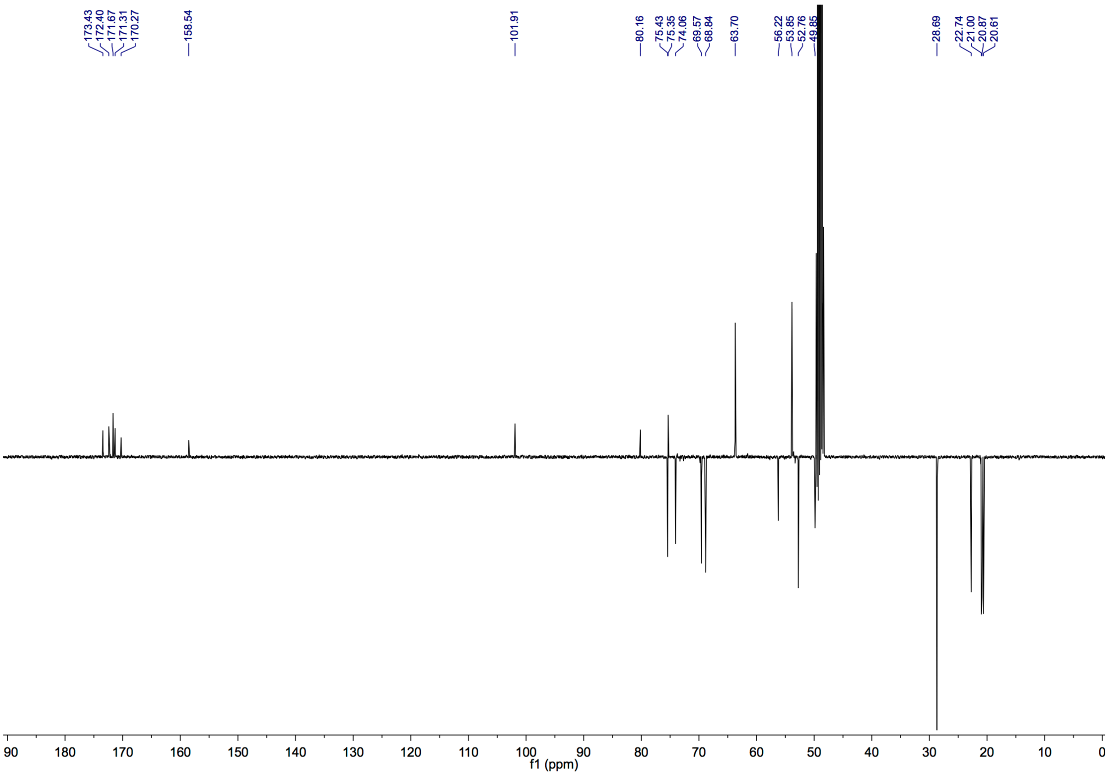

Methyl (prop-2-ynyl 5-acetamido-4-*tert*-butylcarbamido-4,5-dideoxy-D-*glycero*-α-D-*galacto*-non-2-ulopyranosid)onate (**11**)

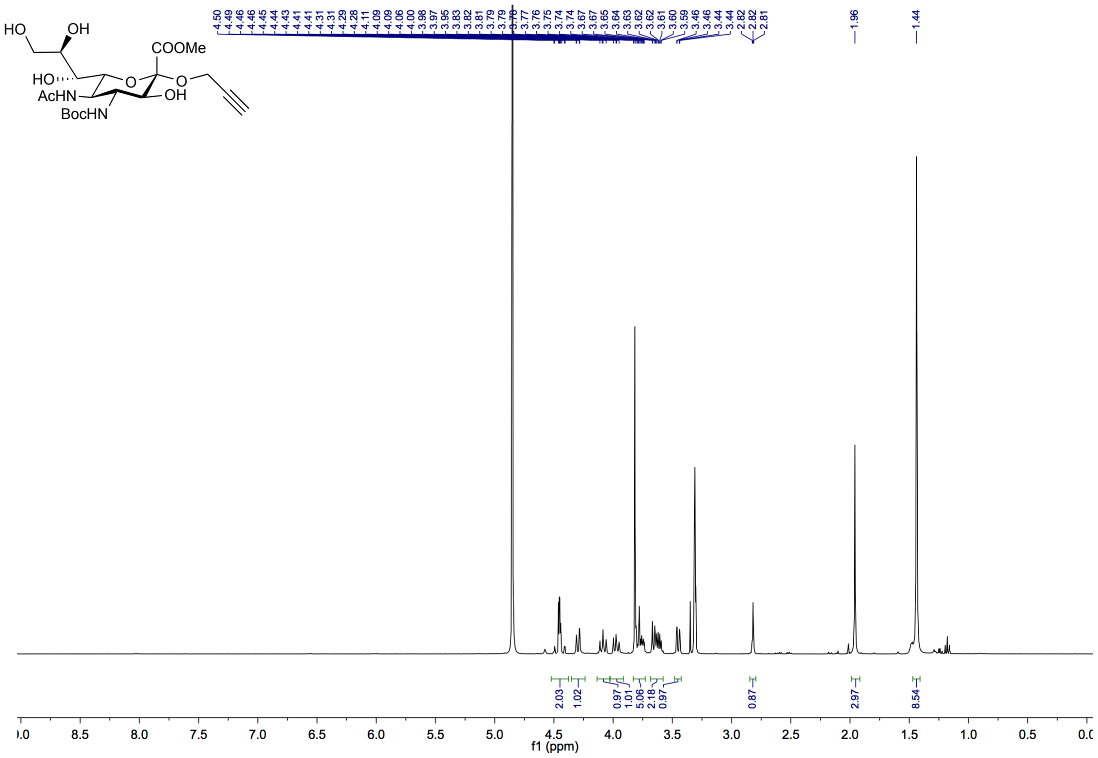

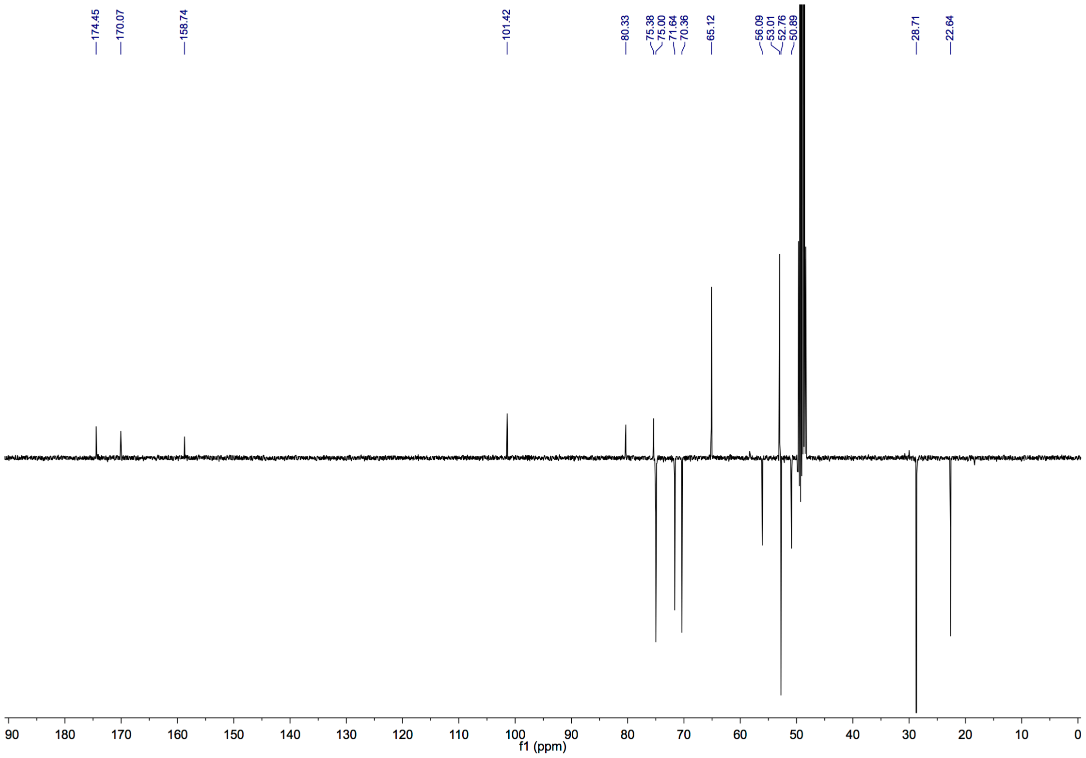

Methyl (prop-2-ynyl 5-acetamido-9-azido-4-*tert*-butylcarbamido-4,5,9-trideoxy-D-*glycero*-α-D-*galacto*-non-2-ulopyranosid)onate (**13**)

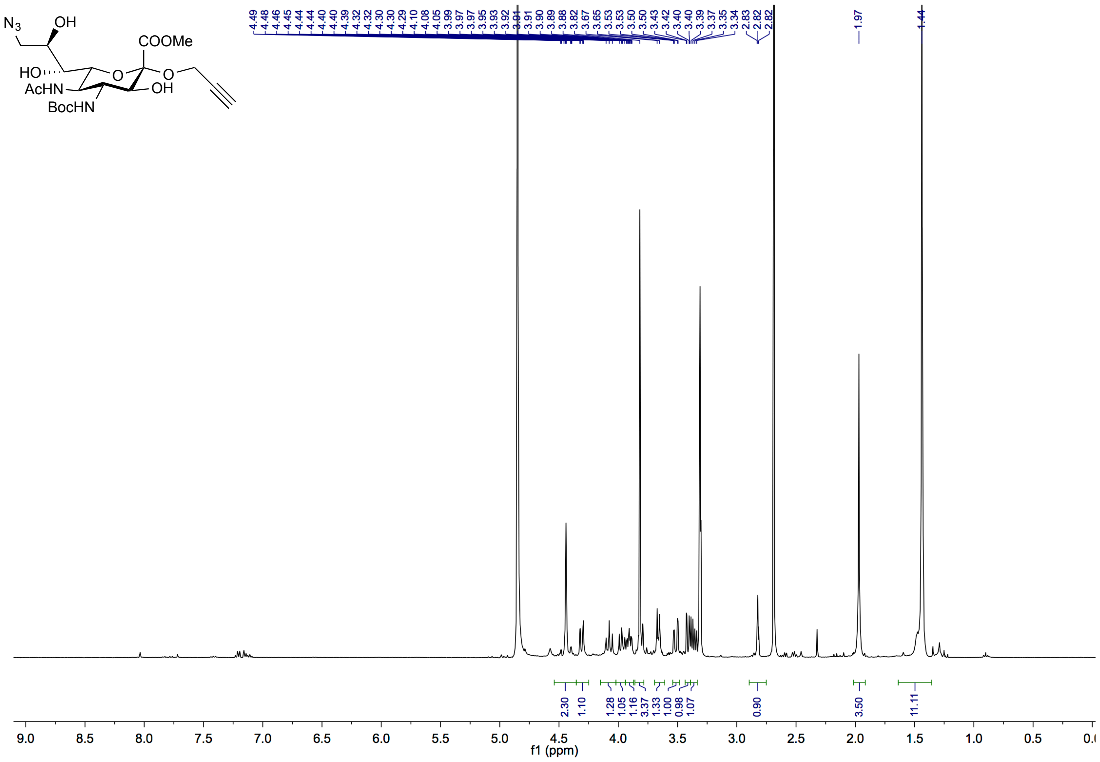

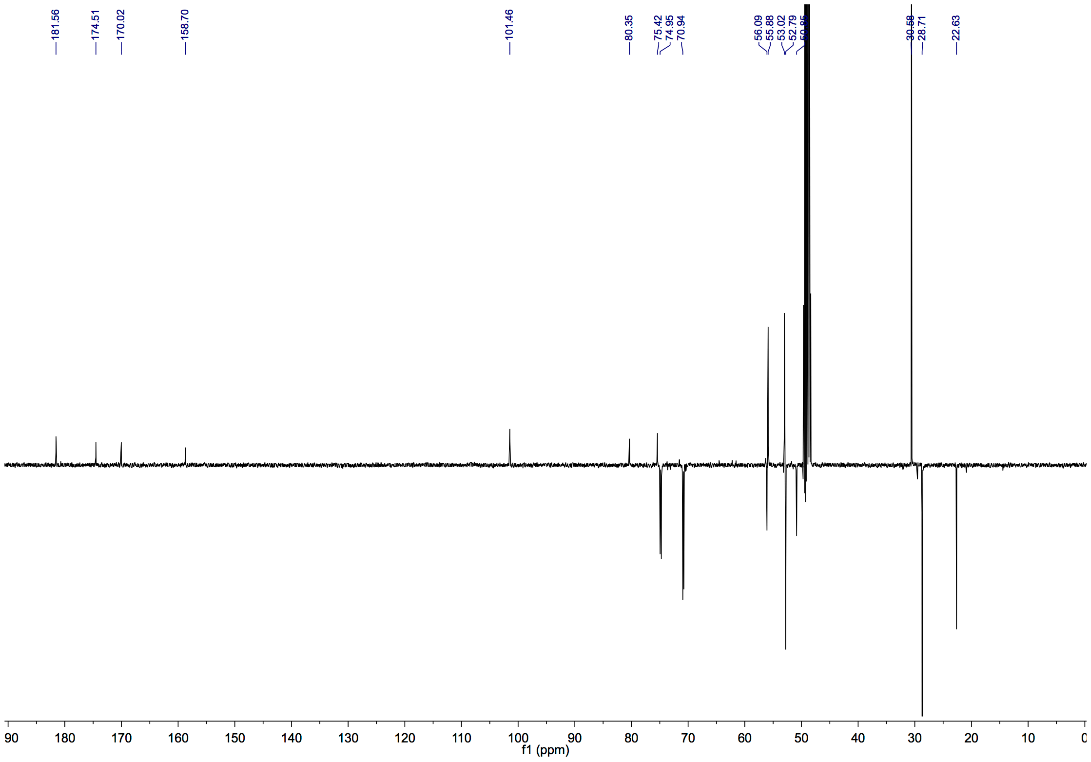

Methyl (prop-2-ynyl 5-acetamido-9-biphenylamido-4-*tert*-butylcarbamido-4,5,9-trideoxy-D-*glycero*-α-D-*galacto*-non-2-ulopyranosid)onate (**14**)

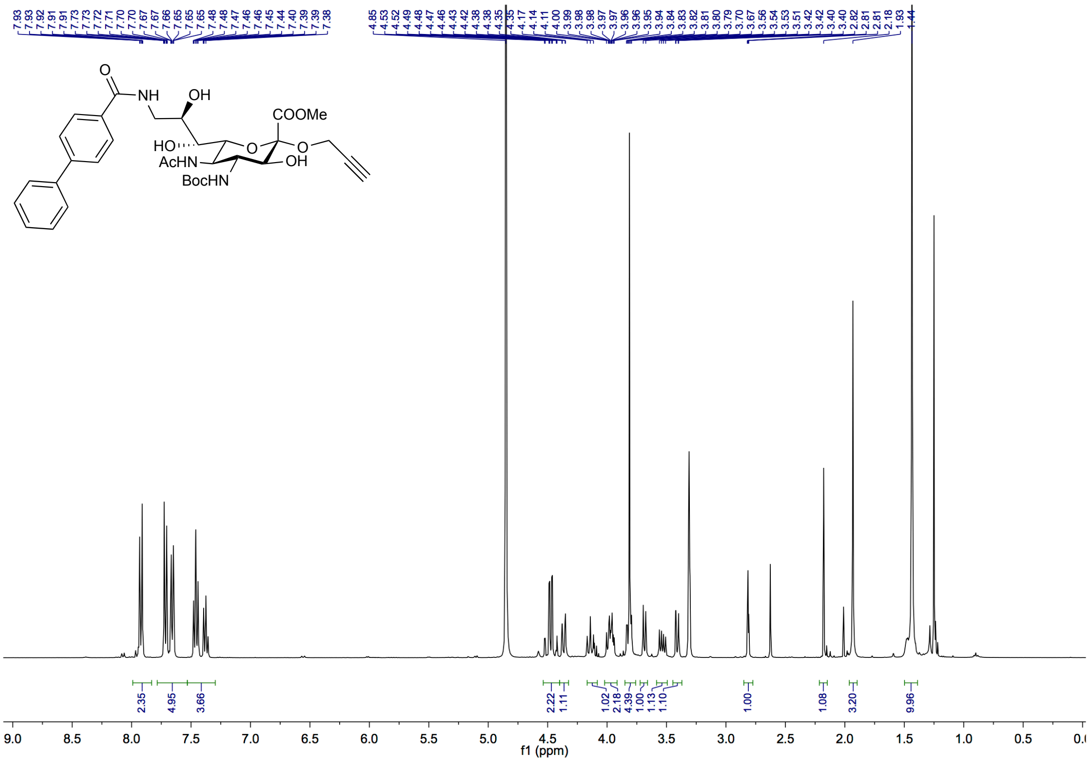

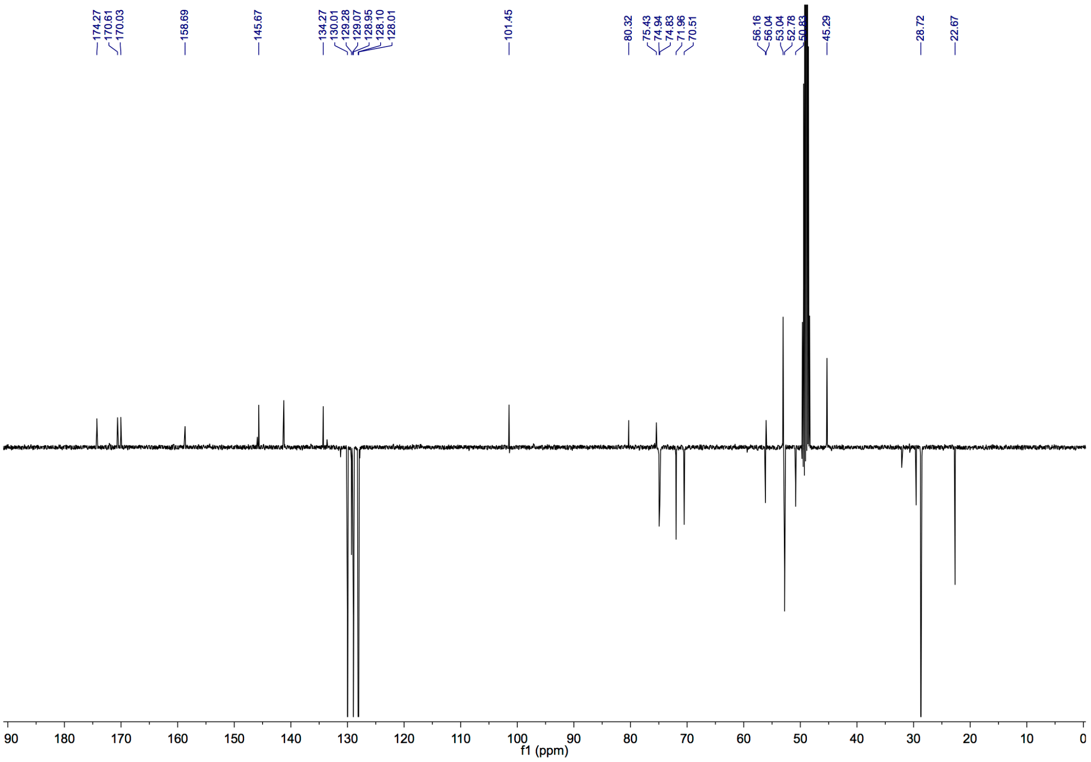

Methyl [prop-2-ynyl 5-acetamido-9-biphenylamido-4-(3'-nitrobenzamido)-4,5,9-trideoxy-D-*glycero*-α-D-*galacto*-non-2-ulopyranosid]onate (**15**)

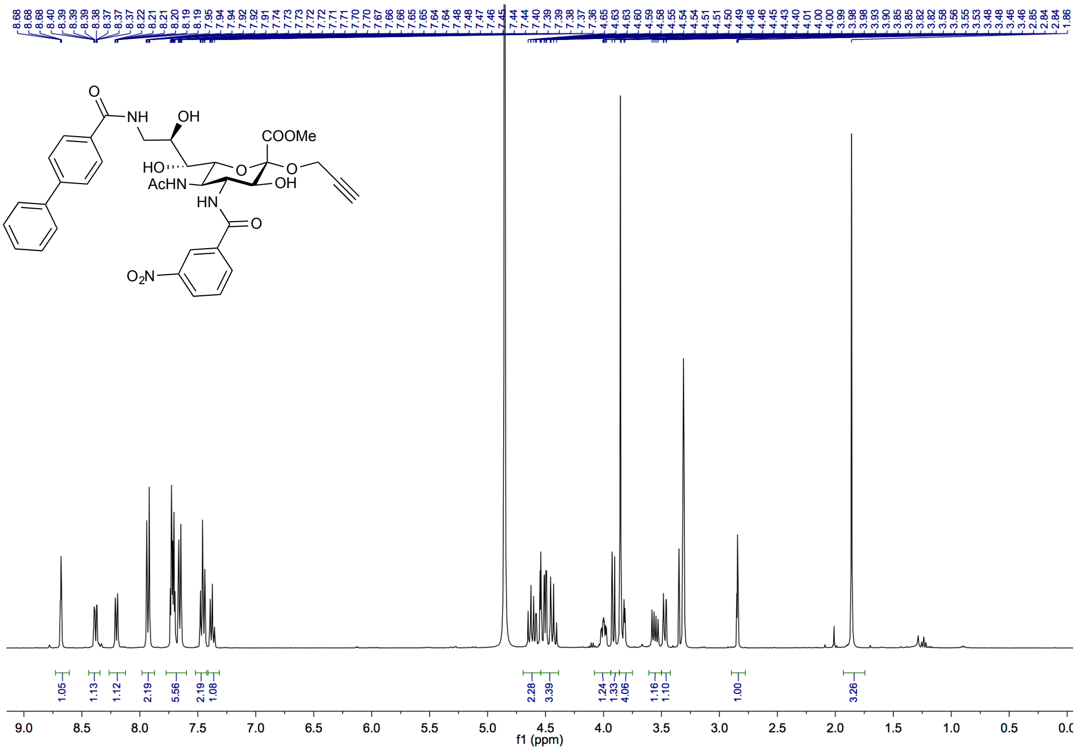

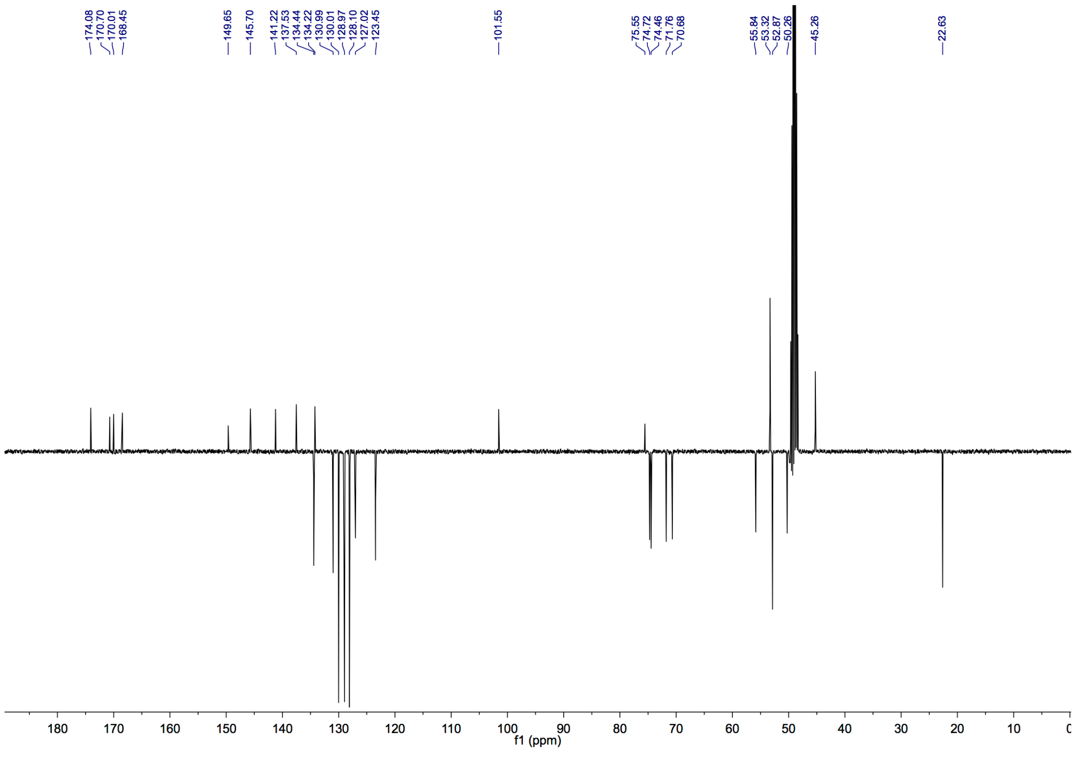

Methyl [prop-2-ynyl 5-acetamido-9-biphenylamido-4-(3'-methoxybenzamido)-4,5,9-trideoxy-D-*glycero*-α-D-*galacto*-non-2-ulopyranosid]onate (**16**)

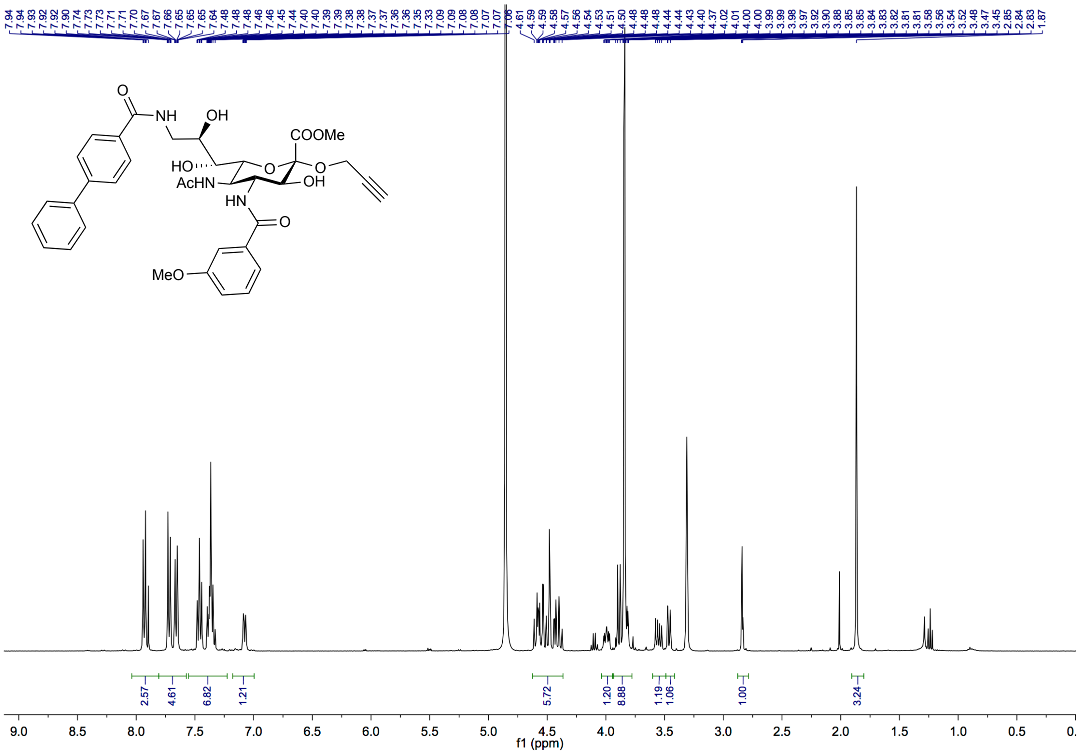

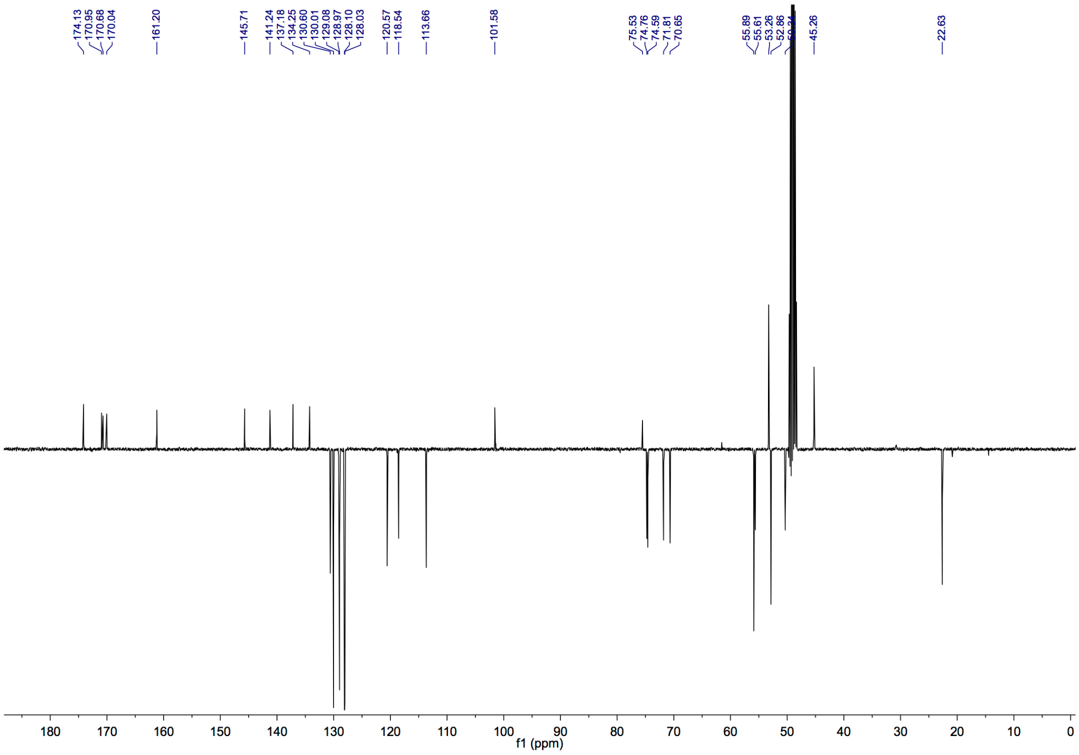

Methyl [(1'-methylacetate-[1',2',3']-triazol-4'-yl)methyl 5-acetamido-9-biphenylamido-4-(3'-nitrobenzamido)-4,5,9-trideoxy-D-*glycero*-α-D-*galacto*-non-2-ulopyranosid]onate (**17**)

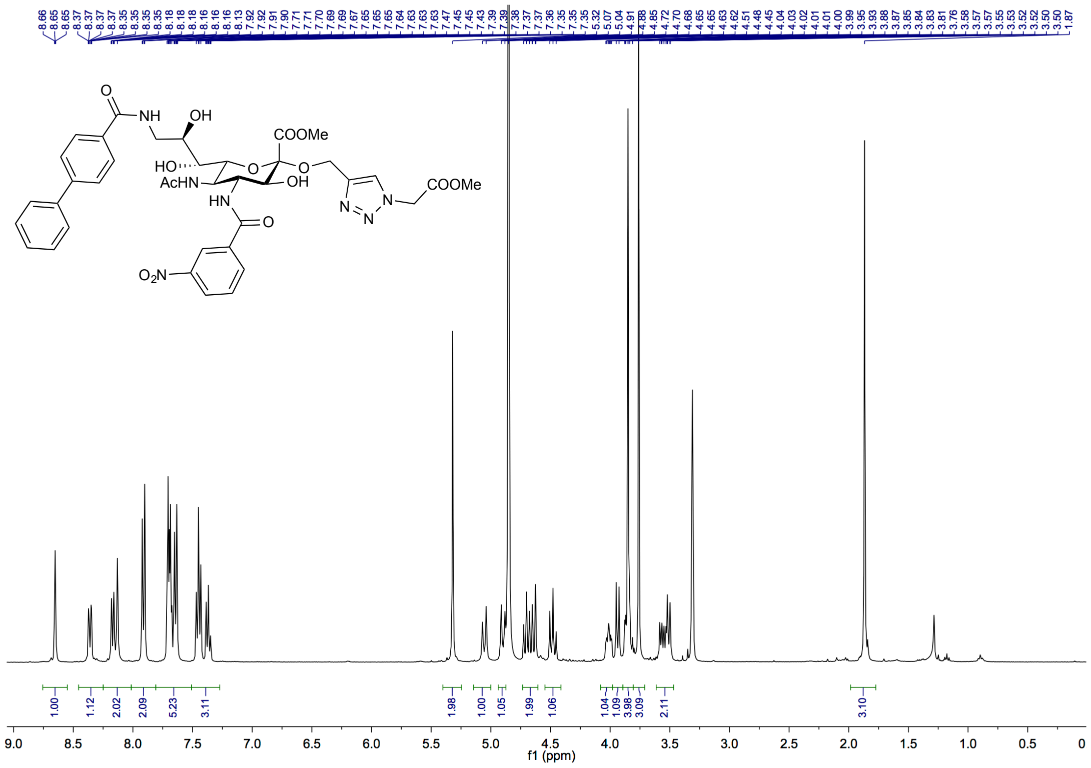

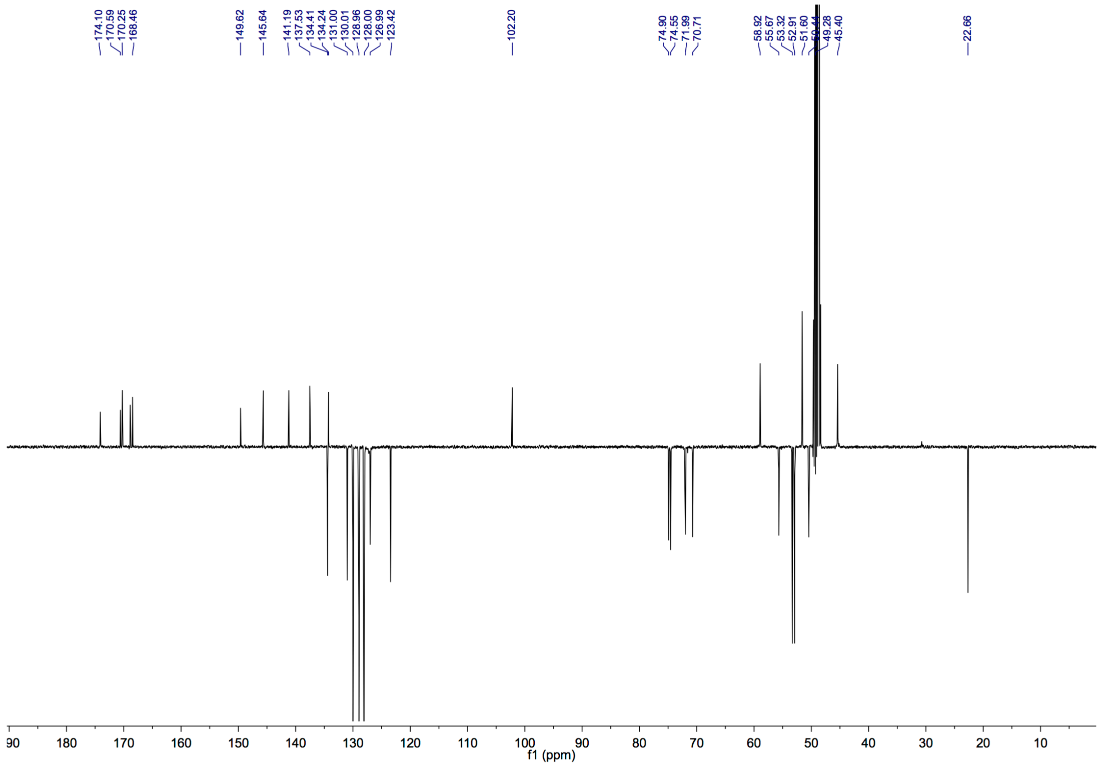

Methyl [(1'-methylacetate-[1',2',3']-triazol-4'-yl)methyl 5-acetamido-9-biphenylamido-4-(3'-methoxybenzamido)-4,5,9-trideoxy-D-*glycero*-α-D-*galacto*-non-2-ulopyranosid]onate (**18**)

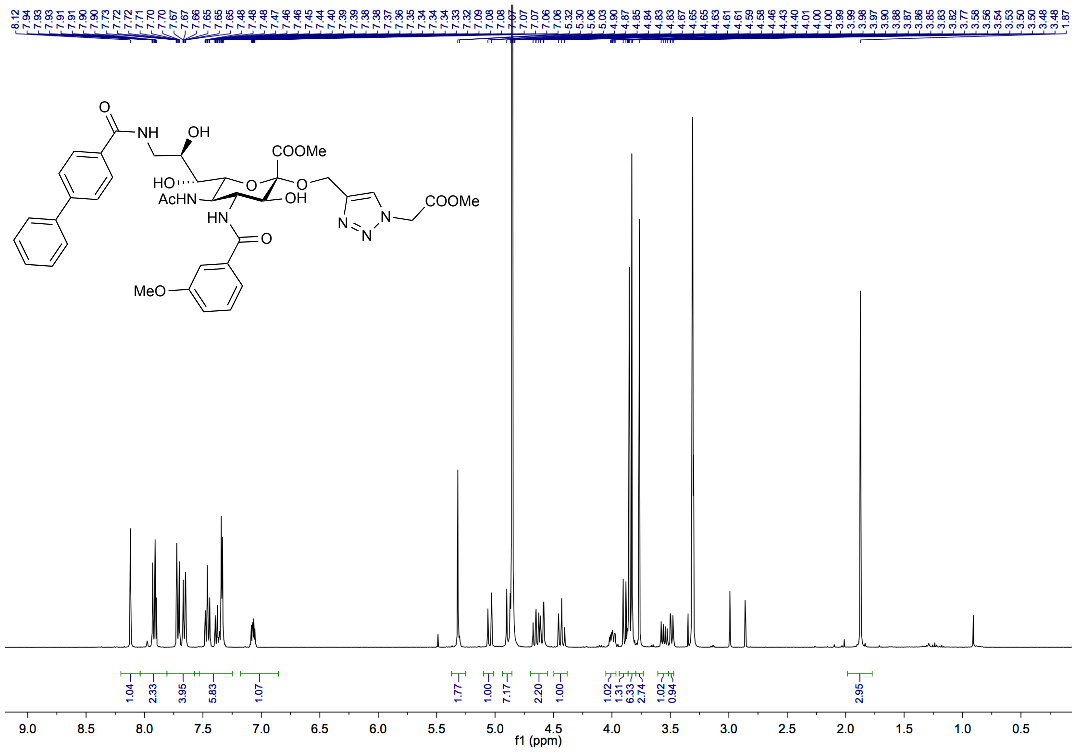

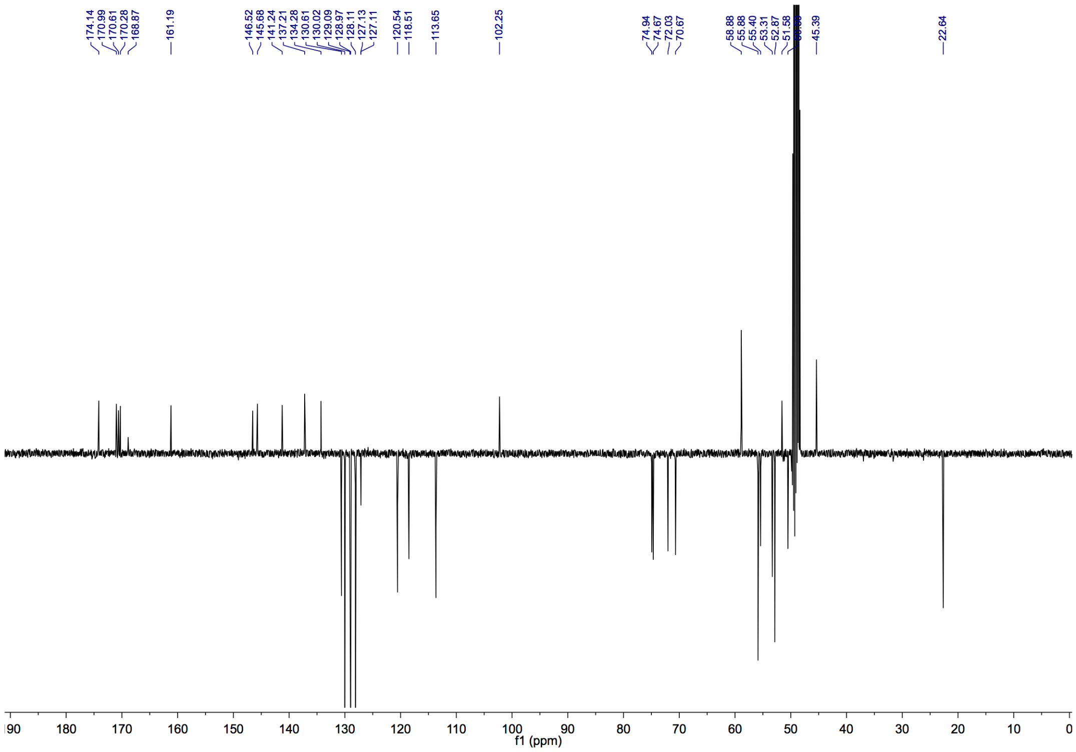

[(1'-carboxymethyl-[1',2',3']-triazol-4'-yl)methyl 5-acetamido-9-biphenylamido-4-(3'-nitrobenzamido)-4,5,9-trideoxy-D-*glycero*-α-D-*galacto*-non-2-ulopyranosidonic acid, disodium salt (**7**)

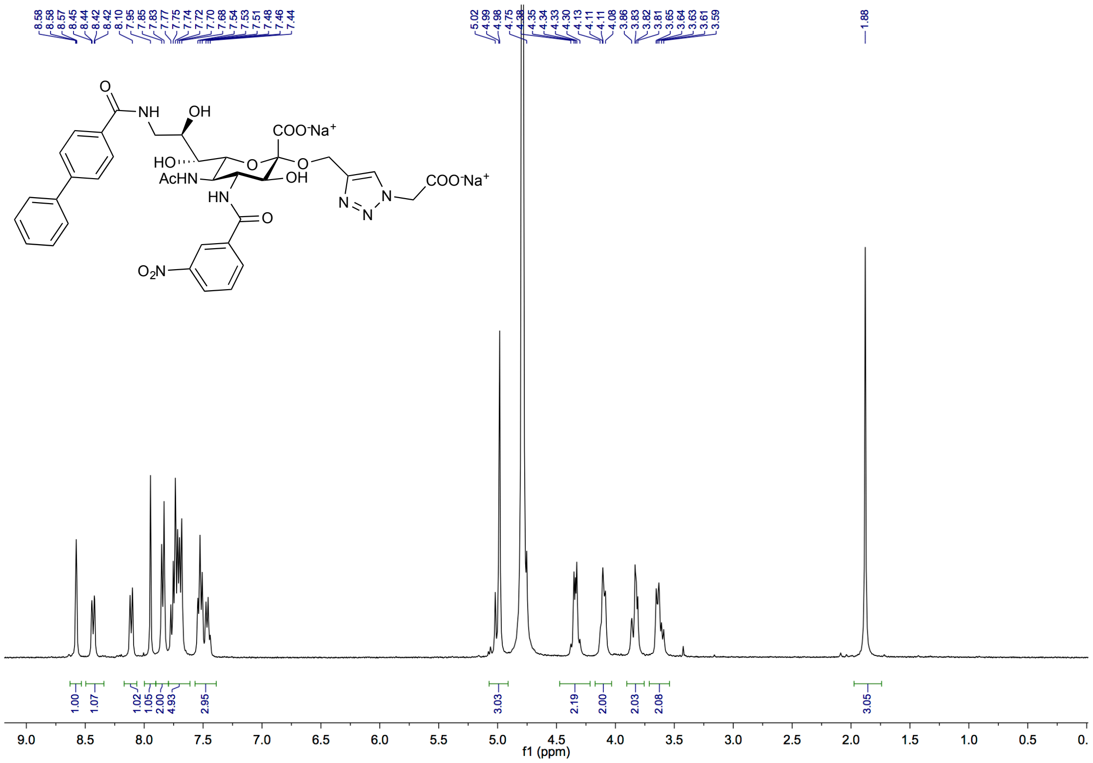

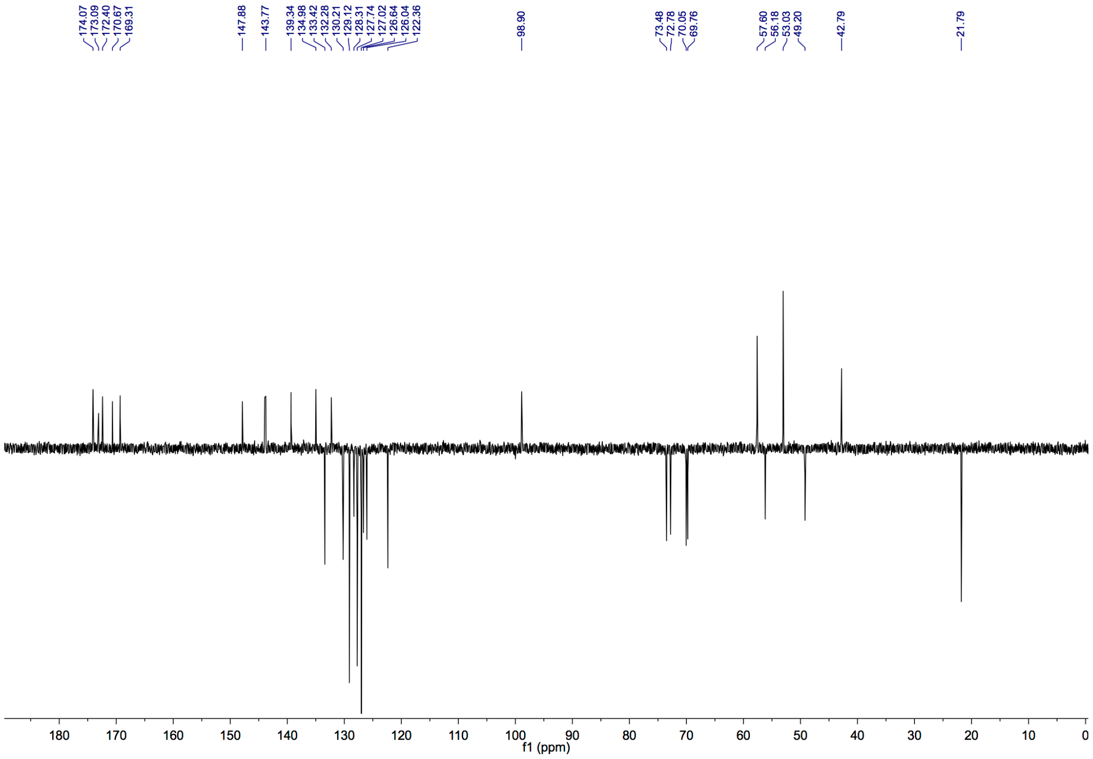

[(1'-carboxymethyl-[1',2',3']-triazol-4'-yl)methyl 5-acetamido-9-biphenylamido-4-(3'-methoxybenzamido)-4,5,9-trideoxy-D-*glycero*-α-D-*galacto*-non-2-ulopyranosidonic acid, disodium salt (**8**)

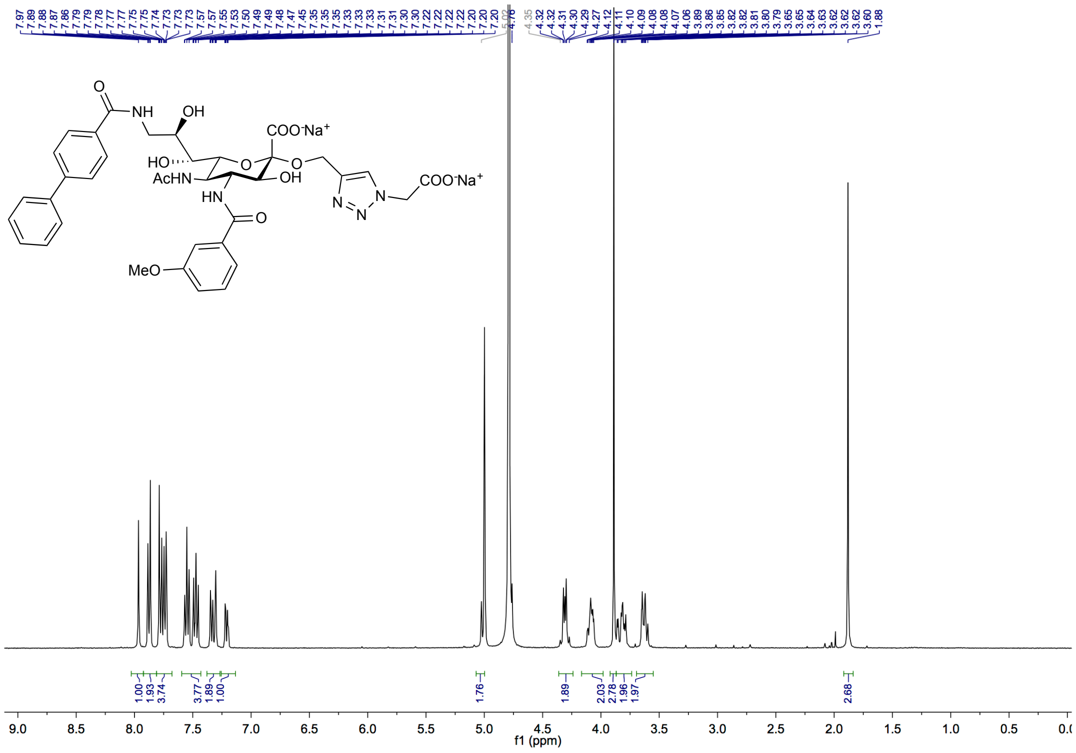

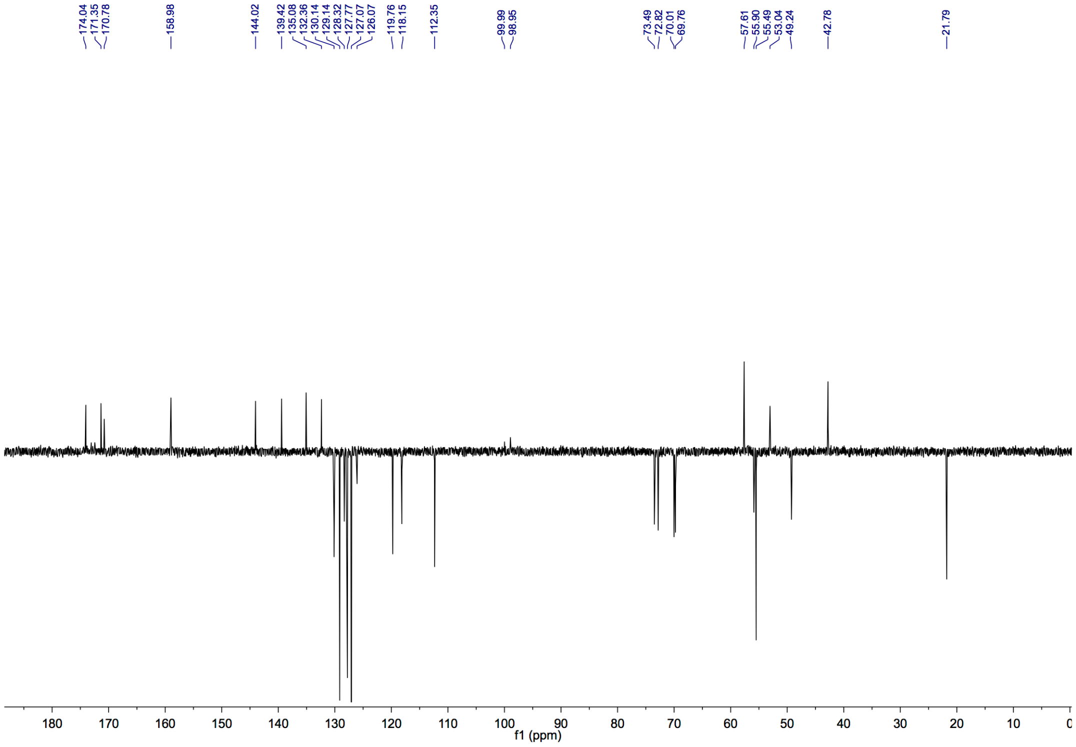

Supplementary references:

1. Schreiner, E.; Zbiral, E.; Kleineidam, R. G.; Schauer, R. *Liebigs Ann. Chem.* **1991**, 129-134.
2. von Itzstein, M.; Jin, B.; Wu, W. Y.; Chandler, M. *Carbohydr. Res.* **1993**, *244*, 181-185.
3. Ciccotosto, S.; von Itzstein, M. *Tetrahedron Lett.* **1995**, *36*, 5405-5408.
4. K. Okamoto, T. Kondo, T. Goto, *Bull. Chem. Soc. Jpn.* **1987**, 60, 631-636.
5. Pascolutti, M., Madge, P., Thomson, R. & von Itzstein, M. *J. of Org. Chem*, **2015**, ***in press***.
6. Kelm, S., Madge, P., Islam, T., Bennett, R., Koliwer-Brandl, H., Waespy, M., von Itzstein, M., and Haselhorst, T. *Angew. Chem., Int. Ed.* **2013,** 52, 3616–3620.
